# Aβ42 promotes the aggregation of α‐synuclein splice isoforms *via* heterogeneous nucleation

**DOI:** 10.1002/1873-3468.70118

**Published:** 2025-07-26

**Authors:** Alexander Röntgen, Zenon Toprakcioglu, Michele Vendruscolo

**Affiliations:** ^1^ Centre for Misfolding Diseases, Yusuf Hamied Department of Chemistry University of Cambridge UK

**Keywords:** Alzheimer's disease, amyloid formation, amyloid‐β, neurodegeneration, Parkinson's disease, α‐Synuclein splice isoforms

## Abstract

Increasing evidence suggests that amyloid‐β (Aβ) and α‐synuclein (αSyn) co‐aggregate in Alzheimer's disease (AD) and Parkinson's disease (PD), an in other neurodegenerative disorders. We investigated how Aβ42 – the predominant Aβ form in AD – co‐aggregates with four αSyn splice isoforms (αSyn‐140, αSyn‐126, αSyn‐112 and αSyn‐98) implicated in PD, finding evidence of a two‐step process. Aβ42 first aggregated into fibrillar assemblies, which then acted as potent nucleation surfaces for initiating the aggregation of αSyn isoforms. Furthermore, pre‐formed Aβ42 seeds promoted αSyn aggregation more strongly than *in situ* Aβ42 aggregates. Our results reveal a unified Aβ–αSyn co‐aggregation mechanism, where Aβ aggregation and αSyn splicing synergistically drive co‐deposition. These findings could help develop therapeutic tools to target key steps in disease‐related co‐aggregation pathways.

**Impact statement**

By demonstrating that Aβ42 fibril seeds serve as potent heterogeneous nucleation surfaces for four common α‐synuclein splice isoforms, this study mechanistically links protein aggregation in Alzheimer's and Parkinson's diseases. Kinetic analysis identifies early cross‐seeding events, suggesting intervention points to delay mixed amyloid pathologies in neurodegeneration.

## Graphical abstract

The aggregation of amyloid‐β (Aβ) and α‐synuclein (αSyn) is associated with Alzheimer's and Parkinson's diseases. This study reveals that Aβ aggregates serve as potent nucleation sites for the aggregation of αSyn and its splice isoforms, shedding light on the intricate interplay between these two pathogenic proteins.

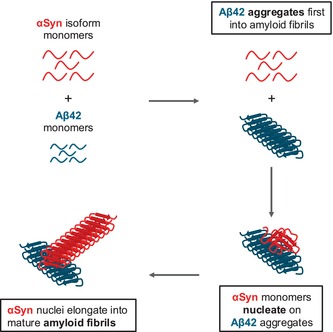

## Abbreviations

**AD**, Alzheimer's disease

**Aβ**, amyloid‐β peptide

**Aβ40**, 40‐residue amyloid‐β peptide

**Aβ42**, 42‐residue amyloid‐β peptide

**DLB**, dementia with Lewy bodies

**ELISA**, enzyme‐linked immunosorbent assay

**PD**, Parkinson's disease

**SD**, standard deviation

**SDS‐PAGE**, sodium dodecyl sulfate–polyacrylamide gel electrophoresis

***t***_**1/2**_, half‐time (time at which a reaction reaches 50% of its maximum signal)

**TEM**, transmission electron microscopy

**ThT**, thioflavin T (amyloid‐binding fluorescent dye)

**αSyn**, α‐synuclein

Neurodegenerative disorders such as Alzheimer's disease (AD) and Parkinson's disease (PD) represent a significant burden on public health, with their incidence expected to increase in the coming decades due to the ageing population [1, 2]. At the molecular level, these diseases are characterised by the aberrant aggregation of proteins in the brain of affected individuals [3, 4, 5]. AD is associated with the aggregation of Aβ into amyloid plaques [6, 7] and PD with the aggregation of α‐synuclein (αSyn) into Lewy bodies [8, 9].

The molecular mechanisms that underlie the aggregation of these proteins have been studied extensively, leading to the formulation of a kinetic framework that involves a series of interconnected microscopic steps [3, 10]. Initially, protein monomers undergo primary nucleation to form growth‐competent seeds. By further addition of monomers, these seeds elongate into amyloid fibrils, which can in turn catalyse the formation of further seeds through secondary nucleation [3, 10]. The spontaneous, or homogeneous, primary nucleation step is typically slow, but can be accelerated by the presence of surfaces, such as lipid membranes or nanoparticles [3, 10, 11, 12, 13]. This heterogeneous nucleation process can also take place by co‐aggregation, where a more insoluble protein may aggregate first and catalyse the aggregation of a less insoluble protein [14, 15, 16].

Consistent with the co‐aggregation mechanism, mounting evidence suggests that Aβ and αSyn form co‐pathologies in various neurodegenerative conditions [17, 18]. The non‐amyloid‐β component (NAC) region of the αSyn sequence was originally identified in Aβ plaques, providing early neuropathological evidence for an interplay between Aβ and αSyn [19]. Since then, αSyn deposition in AD patients as well as Aβ deposition in dementia with Lewy bodies (DLB) and PD patients has been frequently observed [17, 18]. The co‐aggregation process, however, is complex, as underscored by the conflicting results reported regarding the directionality of the effect. Injection or co‐expression of αSyn was found to either inhibit or promote Aβ deposition in amyloid precursor protein (APP)‐transgenic (tg) mice [20, 21, 22, 23], while APP co‐expression in αSyn‐tg mice, the injection of fibrillar αSyn into APP/αSyn‐tg mice, and the administration of Aβ to αSyn‐expressing cells all enhanced αSyn aggregation and inclusion formation [21, 23, 24]. *In vitro*, monomeric Aβ40 and Aβ42 have been shown to interact with monomeric αSyn‐140 [25, 26]. Furthermore, using a chemical kinetics approach, monomeric αSyn‐140 was demonstrated to inhibit the aggregation of Aβ42 by blocking the secondary nucleation step [14], whereas αSyn‐140 fibrils accelerated Aβ40 and Aβ42 aggregation [14, 27]. Conversely, monomeric Aβ42 or pre‐formed Aβ40 and Aβ42 fibrils have been suggested to promote αSyn‐140 aggregation [27, 28], whereas another recent study posited that Aβ42 oligomers, but not monomers or fibrils, cross‐seed αSyn‐140 aggregation [29]. These apparently conflicting results have confounded the issue and point out the lack of a consensus in understanding the interaction of Aβ42 and αSyn aggregation. There is therefore a need to investigate these interactions in a systematic and methodical way.

Contributing further complexity, in addition to the full‐length αSyn‐140 variant, more αSyn isoforms are generated by alternative splicing. These isoforms include αSyn‐126, missing exon 3 (amino acids 41–54), αSyn‐112, missing exon 5 (amino acids 103–130) and αSyn‐98, missing both exons [19, 30, 31, 32, 33]. It has been previously shown that deletion of exon 5, as in αSyn‐112 and αSyn‐98, greatly enhances the aggregation propensity of αSyn and that small amounts of αSyn‐112 mixed with αSyn‐140 significantly accelerate the overall aggregation process, highlighting the need to further characterise the pathological role of αSyn splice isoforms [34].

In this work, we investigated the co‐aggregation of Aβ42 with αSyn and its three splice isoforms to obtain a more complete view of the molecular mechanisms of the interaction. We used biophysical assays paired with kinetic analysis and transmission electron microscopy (TEM) to dissect the microscopic steps in the aggregation of αSyn isoforms affected by Aβ42. Our study unifies previous seemingly contradictory findings about the *in vitro* aggregation of Aβ42 with αSyn, and deepens our understanding of the aggregation propensity and co‐aggregation of αSyn splice isoforms across experimental systems.

## Materials and methods

### Recombinant production and purification of αSyn isoforms

αSyn isoforms (αSyn‐140, αSyn‐126, αSyn‐112, αSyn‐98) were expressed in BL21(DE3) *Escherichia coli* bacteria using the pT7‐7 plasmid (αSyn‐140) or pET29a(+) plasmid (αSyn‐126, αSyn‐112, αSyn‐98) and purified based on previously described protocols [34, 35]. All isoforms were finally eluted in 50 mm Tris/HCl, pH 7.4, snap‐frozen in liquid N_2_ and stored at −80 °C. The molecular identity of the αSyn isoforms was verified by liquid chromatography mass spectrometry (Fig. S1A–D).

### Recombinant production and purification of aβ

Aβ42 and Aβ40 peptides were expressed with an H_6_‐NT fusion tag and a tobacco etch virus protease cleavage site in BL21(DE3) *E. coli* bacteria using the pT7 plasmid and purified based on previously described protocols [36, 37, 38]. Aβ peptides were finally eluted in 50 mm Tris/HCl, pH 7.4, snap‐frozen in liquid N_2_, lyophilised and stored at −80 °C. The molecular identity of the Aβ peptides was verified by liquid chromatography mass spectrometry (Fig. S1E,F).

### Aggregation assays

To assess protein aggregation, αSyn isoforms and Aβ42 peptides were diluted to the required concentration in 50 mm Tris/HCl, pH 7.4, with 50 μm thioflavin T (ThT). The mixtures were transferred onto 96‐well Half‐Area Black with Clear Flat Bottom Polystyrene Non‐Binding Surface Microplates at 100 μL per well. Plates were sealed using Thermowell™ Sealing Tape (Corning Inc, New York, USA) and incubated at 37 °C under quiescent conditions on a FLUOStar Omega plate reader (BMG Labtech, Ortenberg, Germany) to monitor ThT fluorescence intensity over time. For aggregation assays using Aβ42 fibril seeds, Aβ42 was aggregated at a final concentration of 5 μm with 50 μm ThT for 2 days. For aggregation assays using αSyn fibril seeds, αSyn isoforms were aggregated at 50 μm with 50 μm ThT for 5 days [34]. Fibrils were recovered from the plate, collected by centrifugation and resuspended in 50 mm Tris/HCl, pH 7.4. Subsequently, fibrils were sonicated using a Sonopuls HD2070 microtip sonicator (BANDELIN electronic, Berlin, Germany). The resulting Aβ42 seeds were mixed with αSyn isoform monomers, or vice versa, in 50 mm Tris/HCl, pH 7.4, with 50 μm ThT, and aggregation assays were performed as described above.

### Kinetic analysis

All aggregation assays were performed with at least three repeats. Kinetic data were normalised with respect to minimum and maximum ThT fluorescence intensities, and half‐times (*t*_1/2_) were calculated based on the time when ThT fluorescence reached half of its maximum value. Median traces were chosen for representation, and data were then further analysed using the AmyloFit 2.0 platform to determine kinetic rate constants [39].

### SDS/PAGE

A sample of the aggregation mixture at each timepoint was recovered from the aggregation plate and pipetted into a 1.5 mL Protein LoBind tube (Eppendorf, Hamburg, Germany). The mixture was then centrifuged (21 100 ***g***, 20 min, RT), after which the supernatant was transferred to a fresh tube. Supernatant and pellet were each mixed with 4× and 1× NuPAGE LDS sample buffer (ThermoFisher Scientific, Waltham, MA, USA), respectively, and boiled for 5 min at 95 °C. The samples were run on a 10‐well 4–12% BisTris NuPAGE gel with 1× NuPAGE MES SDS Running Buffer and SeeBlue Plus2 Pre‐Stained Protein Standard (ThermoFisher Scientific) as the molecular weight marker. The gels were recovered, stained with InstantBlue Coomassie Protein Stain (Abcam, Cambridge, UK), and imaged using the ChemiDoc MP Imaging System (Bio‐Rad, Hercules, CA, USA).

### Transmission electron microscopy

Samples were recovered from aggregation plates and were prepared on carbon films on 3‐mm 300‐mesh copper grids (EM Resolutions Ltd., Keele, UK), which were glow‐discharged using a GloQube Plus (Quorum Technologies, Laughton, UK). 3 μL of sample were spotted on the grids for 40 s and blotted using a Whatman filter paper. The samples were then negatively stained with 3 μL 2% (w/v) uranyl acetate for 40 s and finally air‐dried. TEM images were acquired on an FEI Talos F200X G2 transmission electron microscope (Thermo Fisher Scientific, Waltham, MA, USA).

## Results

### The aggregation of αSyn isoforms is promoted by Aβ42

**Fig. 1:**
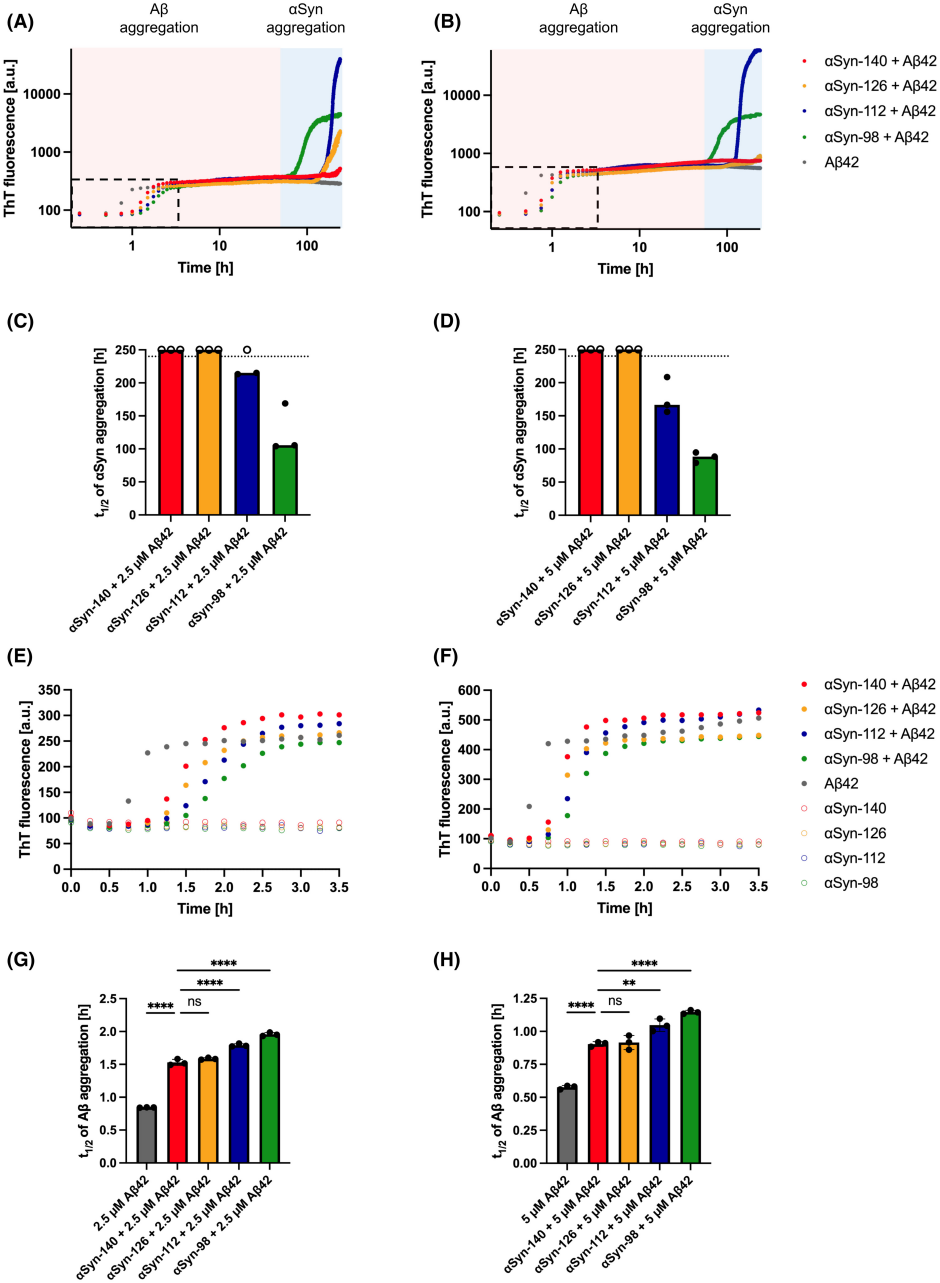
Aggregation of αSyn isoforms in the presence of Aβ42 monomers. (A, B) The aggregation of αSyn isoforms with 2.5 μm (A) and 5.0 μm (B) of Aβ42 monomers at a total protein concentration of 50 μm was assessed over time by monitoring ThT fluorescence intensity. (C, D) Half‐times (*t*_1/2_) of the aggregation traces shown in (A, B), respectively. Data are shown as medians (bars) of three replicates (circles). If the aggregation reaction was completed, half‐times are shown as closed circles; otherwise, replicates are displayed as open circles at a value higher than the total run time. (E, F) Magnification of the first 3.5 h of the aggregation reactions, indicated by the dashed rectangle in (A, B), respectively, showing the inhibition of Aβ42 aggregation by αSyn monomers before reaching the initial plateau phase. (G, H) Half‐times (*t*_1/2_) of Aβ42 aggregation shown in (E, F), respectively. Data are shown as mean ± SD of three replicates. One‐way ANOVA with Šídák's multiple comparisons test. ****P < 0.0001, **P < 0.01, ns, non‐significant.

We first investigated the effects of mixing monomeric Aβ42 with each of the four different αSyn splice isoforms (αSyn‐140, αSyn‐126, αSyn‐112, αSyn‐98). To do this, we used 2.5 or 5.0 μm monomeric Aβ42 with αSyn splice isoforms to reach a total protein concentration of 50 μm and performed kinetic assays using the amyloid‐binding dye thioflavin T (ThT) (Fig. 1). All samples containing Aβ42 and the αSyn isoforms, as well as control samples containing only Aβ42 but no αSyn isoforms, exhibited a minor initial increase in ThT fluorescence after about 1 h (Fig. 1A,B). In contrast, this initial increase was absent in samples containing only αSyn isoforms but no Aβ42 (Fig. 1E,F and Fig. S2). We therefore ascribed this minor increase in ThT fluorescence intensity to the initial aggregation of Aβ42, which is consistent with previous reports on the timescale of Aβ42 aggregation at similar concentrations [40, 41].

In samples containing Aβ42 with αSyn‐112 or αSyn‐98, this initial increase in ThT fluorescence with a short lag time was followed by a marked increase with a long lag time due to the subsequent aggregation of these two αSyn isoforms (Fig. 1A,B). Comparing the half‐times of these reactions reveals that the presence of Aβ42 accelerates αSyn aggregation, here observed for αSyn‐112 (*t*_1/2_ > 240 h without Aβ42, as opposed to 215 h with 2.5 μm Aβ42 and 167 h with 5.0 μm Aβ42) and αSyn‐98 (*t*_1/2_ = 122 h without Aβ42 as opposed to 106 h with 2.5 μm Aβ42 and 89 h with 5.0 μm Aβ42) (Fig. 1C,D and Figs S2 and S3A,B). Moreover, these data corroborate previous findings showing that αSyn‐112 and αSyn‐98, which lack the highly negatively charged sequence region corresponding to exon 5, are more aggregation‐prone compared to αSyn‐140 and αSyn‐126 [34]. Hence, by mixing Aβ42 monomers with αSyn isoforms, we observed a biphasic aggregation reaction, in which Aβ42 monomers first undergo aggregation reaching an initial plateau phase, and subsequently, these Aβ42 aggregates catalyse αSyn aggregation.

To add another level of complexity, it was previously demonstrated that αSyn monomers slow down the aggregation of Aβ42 monomers by binding to Aβ42 fibrils formed *in situ*, thereby inhibiting secondary nucleation of Aβ42 [14]. We therefore further magnified our data within the first 3.5 h to focus on the aggregation kinetics of Aβ42 (Fig. 1E,F and Fig. S3C,D). We thus found that monomers of all αSyn isoforms tested here significantly inhibited Aβ42 aggregation, which is in accordance with previously reported findings for αSyn‐140 [14]. Thus, it can be rationalised that αSyn monomers bind to Aβ42 fibrils formed *in situ*, initially slowing down Aβ42 aggregation. However, on longer timescales as monitored in this study, αSyn isoforms adsorbed to Aβ42 fibrils undergo surface‐catalysed nucleation and thus amyloid aggregation. We found that αSyn‐112 and αSyn‐98 delayed Aβ42 aggregation more strongly than αSyn‐140 and αSyn‐126, as shown by the half‐time analysis (Fig. 1G,H). This effect may be due to stronger binding of αSyn‐112 and αSyn‐98 monomers to Aβ42 fibrils and would be in line with the faster nucleation of these isoforms.

To further test the potency of Aβ42 on the aggregation of αSyn isoforms, we complemented the above experiments by investigating a lower range of Aβ42 concentrations. For this, we incubated 50 μm αSyn‐126, αSyn‐112 and αSyn‐98 with 1.0, 0.5 or 0.1 μm Aβ42 monomers (Fig. S4). Consistent with the results at 2.5 and 5.0 μm Aβ42 (Fig. 1A–D), our analysis confirmed that αSyn‐98 was the fastest‐aggregating isoform under all conditions tested (*t*_1/2_ = 73 h with 1.0 μm Aβ42, 90 h with 0.5 μm Aβ42, and 88 h with 0.1 μm Aβ42) (Fig. S4B,D,F). αSyn‐112 exhibited a similar aggregation half‐time at 1.0 μm (*t*_1/2_ = 209 h) as with 2.5 μm Aβ42 (*t*_1/2_ = 215 h). Of note, the aggregation of αSyn‐112 was fastest in the presence of 0.5 μm Aβ42 (*t*_1/2_ = 155 h), but occurred on a markedly prolonged timescale with 0.1 μm Aβ42 (*t*_1/2_ = 310 h) (Fig. S4B,D,F). The αSyn‐126 isoform, which had not exhibited full aggregation at higher Aβ42 concentrations (Fig. 1A,B and Fig. S3A,B), appeared to have an aggregation optimum at 1.0 μm Aβ42 (*t*_1/2_ = 179 h) and also readily aggregated in the presence of 0.5 μm (*t*_1/2_ = 188 h) and 0.1 μm Aβ42 (*t*_1/2_ = 215 h) (Fig. S4B,D,F). Furthermore, we repeated the control conditions of incubating 50 μm αSyn isoforms without adding any Aβ42 to cover the extended timescale of this experiment (Fig. S4G,H). We found that αSyn‐98 again aggregated first (*t*_1/2_ = 119 h), which was followed by αSyn‐126 (*t*_1/2_ = 314 h). In contrast, no aggregation was observed for αSyn‐112 in the absence of Aβ42 (*t*_1/2_ > 375 h). Hence, these results confirm our prior hypothesis that Aβ42 accelerates the aggregation of αSyn isoforms. Importantly, we found that the aggregation of αSyn isoforms can be promoted by low micromolar and even nanomolar concentrations of Aβ42, which is in line with the concentrations estimated to be present within neurons recovered from AD patients [42]. This finding is critical, as previous work has typically focused on micromolar Aβ concentrations, which are at least an order of magnitude higher than physiological Aβ concentrations. The fact that, even at nanomolar concentrations, we observed this co‐aggregation behaviour shows that Aβ can potently influence αSyn aggregation.

To further expand our understanding of the interaction between αSyn isoforms and Aβ, we next sought to assess whether a similar effect, that is the acceleration of αSyn isoform aggregation, could be achieved using the less aggregation‐prone Aβ40 variant. We therefore incubated each of the four αSyn isoforms at 50 μm with 5.0 μm Aβ40 monomers and, as before, monitored the aggregation using ThT (Fig. S5). Analogous to the experiment with Aβ42, two phases of aggregation were observed. The first phase of aggregation coincided with the Aβ40‐only control, which, as expected, took place on a much longer timescale compared to Aβ42. The Aβ40 variant is well known to aggregate significantly more slowly than Aβ42 due to the lack of the two hydrophobic, C‐terminal amino acids, isoleucine and alanine. Following the initial aggregation phase, we observed again a second phase of aggregation, which can be attributed to the aggregation of αSyn isoforms (Fig. S5A). The half‐times of these traces are shown in Fig. S5B. As before with Aβ42, we further determined the half‐times of the initial Aβ40 aggregation in the presence and absence of αSyn isoforms (Fig. S5C,D). Contrary to the results with Aβ42, which had revealed a clear trend whereby αSyn‐140 through αSyn‐98 progressively inhibited the initial Aβ aggregation (Fig. 1E–H), such a trend was absent with Aβ40 (Fig. S5D). It rather appears that the change in Aβ40 aggregation half‐time strongly varies depending on each αSyn isoform that is incubated with Aβ40.

Taken together, the results of the investigations of mixtures of αSyn with Aβ show that the presence of Aβ catalyses the aggregation of αSyn isoforms, which, except for the most aggregation‐prone αSyn‐98 isoform, is otherwise not observed before 250 h under the conditions tested. Moreover, our experiments reveal that the nucleation speed of αSyn isoforms is sensitive to the isoform‐specific, molecular interactions between αSyn and Aβ, fine‐tuning the aggregation of each of the αSyn variants. The overall aggregation speed therefore likely depends on an interplay of several molecular events, including the inhibition of Aβ aggregation by αSyn, thus affecting the *in‐situ* formation of nucleation sites for αSyn aggregation, followed by the adsorption of αSyn monomers to Aβ aggregates, the formation of primary αSyn nuclei, their desorption from Aβ aggregates into the reaction mixture and elongation by addition of further αSyn monomers.

### Pre‐formed Aβ42 fibrils promote the aggregation of the αSyn isoforms more efficiently than Aβ42 monomers

**Fig. 2:**
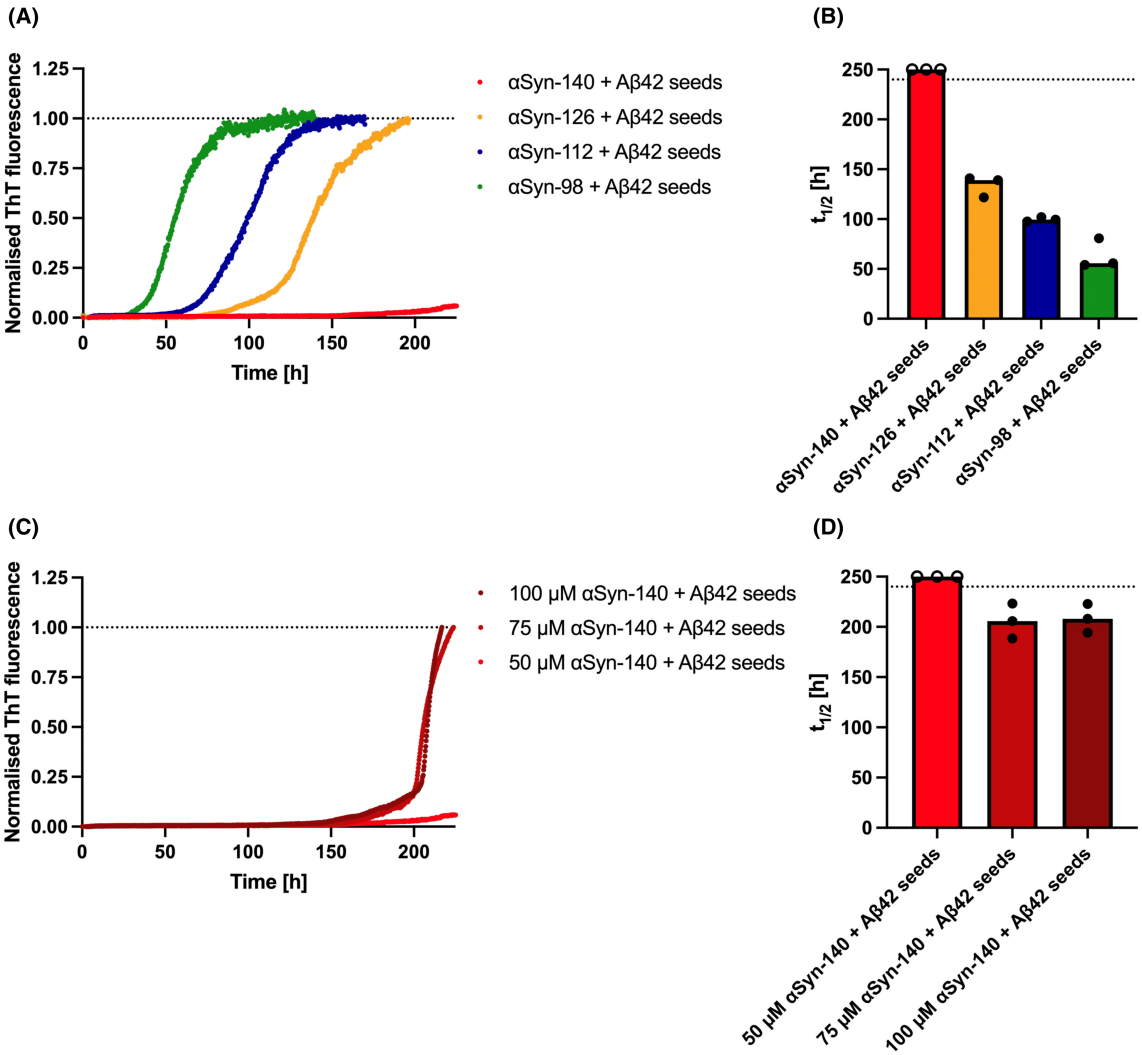
Aggregation of αSyn isoforms in the presence of Aβ42 seeds. (A, C) The aggregation of 50 μm αSyn isoforms with 5.0 μm Aβ42 seeds was assessed over time by monitoring ThT fluorescence intensity. (B, D) Half‐times (*t*_1/2_) of the aggregation traces shown in (A, C), respectively. Data are shown as medians (bars) of three replicates (circles). If the aggregation reaction was completed, half‐times are shown as closed circles; otherwise, replicates are displayed as open circles at a value higher than the total run time. Data for 50 μm αSyn‐140 in (A, C) are identical since they were assessed together for comparison.

Given the observation of a biphasic aggregation behaviour when mixing Aβ42 monomers with αSyn isoform monomers, we hypothesised that adding pre‐formed Aβ42 fibril seeds (denoted as seeds henceforth) to αSyn isoform monomers should result in a monophasic aggregation behaviour (since Aβ42 is already pre‐aggregated) and further shorten the time for αSyn isoforms to undergo aggregation (Fig. 2). To explore this, we mixed 5.0 μm Aβ42 seeds with 50 μm αSyn isoform monomers and observed the aggregation of αSyn‐98 (*t*_1/2_ = 56 h), followed by αSyn‐112 (*t*_1/2_ = 100 h), and αSyn‐126 (*t*_1/2_ = 139 h) (Fig. 2A,B). The latter was not observed to fully aggregate before, when mixing αSyn‐126 with Aβ42 monomers (Figs 1A,B). Furthermore, the aggregation half‐times were reduced by ~40% for αSyn‐112 and αSyn‐98 with Aβ42 seeds compared to mixtures with Aβ42 monomers (Figs 1D and 2B). Thus, these results confirm that pre‐formed Aβ42 seeds are more efficient at promoting the aggregation of αSyn isoforms than Aβ42 monomers. This finding is also consistent with the conclusion above that Aβ42 monomers first need to undergo aggregation *in situ* to serve as nucleation sites for αSyn isoforms.

Again, the aggregation of the main isoform αSyn‐140 was not observed under the given conditions. Hence, we sought to compare mixtures containing a constant amount of 5.0 μm Aβ42 seeds with increasing amounts of αSyn‐140 (50, 75 and 100 μm) (Fig. 2C) to assess at which concentration αSyn‐140 may undergo amyloid aggregation. Indeed, aggregation was observed at both 75 μm (*t*_1/2_ = 206 h) and 100 μm αSyn‐140 (*t*_1/2_ = 208 h) (Fig. 2C,D). These results show that αSyn‐140 is the least aggregation‐prone out of the four αSyn isoforms characterised in this study, and it demonstrates that deletions of exon 3 (αSyn‐126), exon 5 (αSyn‐112) and both exons 3 and 5 (αSyn‐98) progressively enhance the aggregation propensity of αSyn in the presence of Aβ42.

Based on the above‐shown aggregation experiments using ThT, we conclude that Aβ42 aggregates, either formed from monomers *in situ*, or added as pre‐formed seeds, promote the aggregation of αSyn isoforms. This mechanism is supported by the fact that the aggregation traces of mixtures between αSyn isoforms and Aβ42 monomers are clearly divided into two phases — a first, early aggregation phase coinciding with the aggregation of the Aβ‐only control, followed by a second, late aggregation phase, which we ascribe to αSyn aggregation (Fig. 1A,B and Fig. S5A,C). In contrast, the initial increase in ThT fluorescence is absent when Aβ42 seeds instead of monomers are added (Fig. 2A,C). Importantly, αSyn aggregation is not observed at all before 250 h of incubation if no Aβ42 is added (except for the most aggregation‐prone variant αSyn‐98) (Figs S2 and S4G,H).

**Fig. 3:**
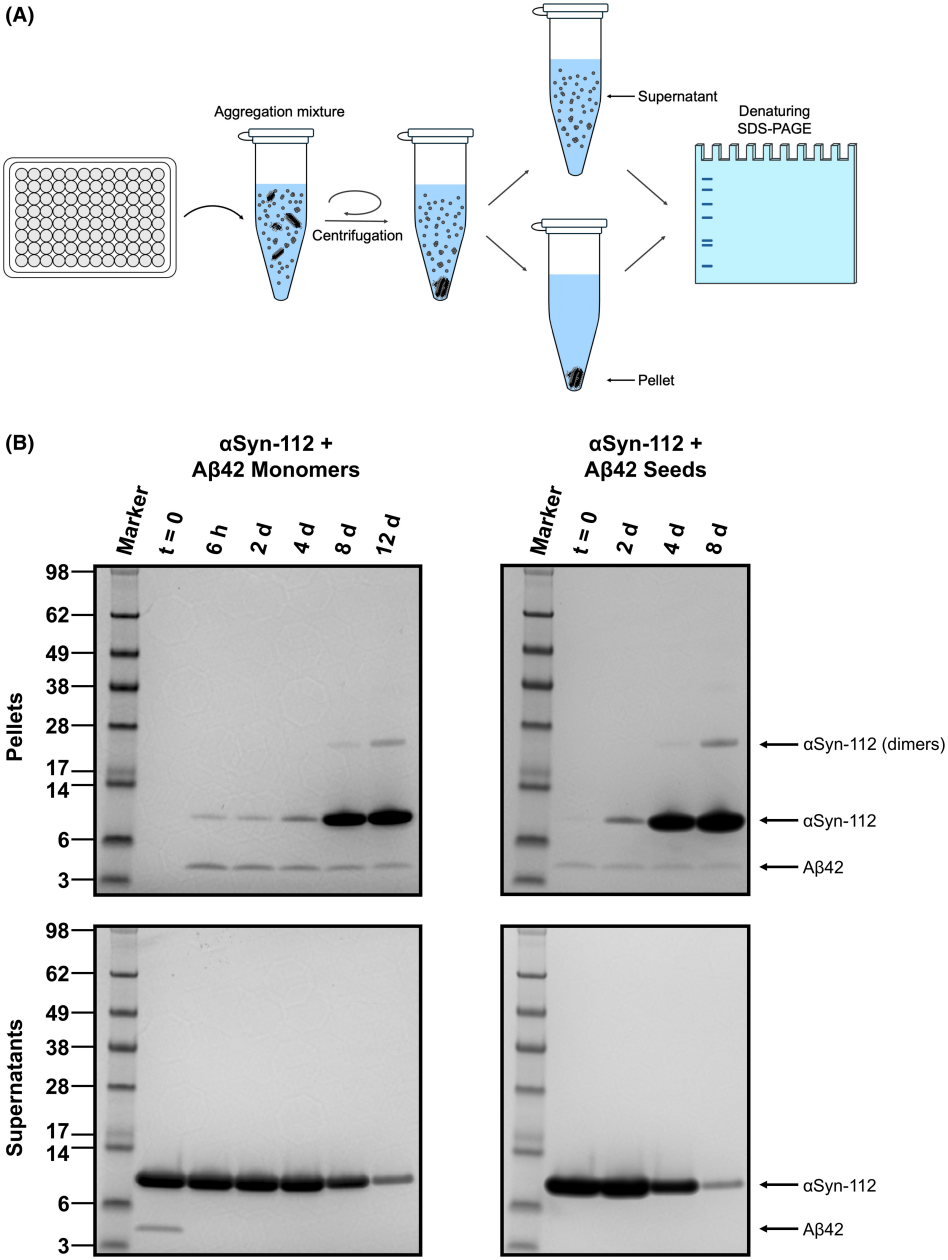
Tracing of the co‐aggregation reaction using SDS‐PAGE. (A) Schematic representation of the workflow. 50 μm αSyn‐112 monomers were mixed with either 5 μm Aβ42 monomers or 5 μm Aβ42 seeds and incubated on a plate reader. The aggregation mixtures were recovered from the aggregation plate at different time points and centrifuged to separate insoluble, aggregated species (pellet) vs. soluble species (supernatant). The content of both fractions was denatured, separated by size using SDS‐PAGE, and visualised using Coomassie stain. (B) Gel images of the aggregation reaction of αSyn‐112 monomers with Aβ42 monomers (left panels) and αSyn‐112 monomers with Aβ42 seeds (right panels). Molecular weights are displayed as kilodaltons.

To verify this mechanism of aggregation, and in particular to identify the species that aggregate in each of the observed phases, that is the first or second phase, we used an SDS‐PAGE‐based approach. To this end, we used αSyn‐112 because we found in the previous experiments that this isoform readily aggregates in the presence of Aβ42 but does not aggregate in the absence of Aβ42 (Figs 1A‐D, 2A,B, S2 and S4G,H). Thereby, we ensured that the aggregation we observe with this variant is entirely related to the presence of Aβ42. We then mixed 50 μm αSyn‐112 with either 5 μm Aβ42 monomers or 5 μm Aβ42 seeds and analysed samples of the aggregation mixture at various timepoints along the aggregation reaction. The workflow we employed is schematically depicted in Fig. 3A. At any given timepoint, a sample of the aggregation mixture was recovered from the plate and immediately centrifuged to pellet any insoluble, aggregated species, whereas soluble species remained in the supernatant. We then separated the supernatant from the pellet and denatured the contents of both fractions using dodecyl sulfate. The species present in either of the two fractions were then separated according to their size using SDS‐PAGE and visualised by Coomassie staining. The corresponding gel images are shown in Fig. 3B, and the results from this approach are in full agreement with the ThT aggregation traces shown above (Figs 1A,B and 2A). When mixing αSyn‐112 with Aβ42 monomers (Fig. 3B, left panels), both species were found entirely in the supernatant at the start of the reaction (*t* = 0), and none in the pellets, confirming that neither αSyn‐112 nor Aβ42 had aggregated yet. After completion of the first phase of aggregation, that is at 6 h, all Aβ42 was pelleted by centrifugation, indicating that Aβ42 had entirely assembled into aggregates. In contrast, only a very small portion of αSyn‐112 was found in the pellet, whereas most of the protein remained in the supernatant. At subsequent timepoints, the amount of aggregated αSyn‐112 in the pellet steadily increased while the amount of soluble αSyn‐112 in the supernatant accordingly decreased. The largest increment of aggregated αSyn‐112 found in the pellet was observed at 8 days, which is in line with the half‐time of the aggregation reaction measured by ThT and thus in the exponential phase of aggregate formation (Fig. 1D). At the two latest timepoints (8 and 12 days), αSyn‐112 dimers were also observed on the gel. These appear to be resistant to denaturation as the content of aggregated αSyn‐112 approaches its maximum.

Likewise, we conducted this experiment with αSyn‐112 monomers mixed with Aβ42 seeds (Fig. 3B, right panels). At the start of the reaction (*t* = 0), Aβ42 was entirely found in the pellet after centrifugation, which is expected given that Aβ42 is added in the form of pre‐aggregated seeds. A very faint band of αSyn‐112 can also be noted, likely representing αSyn‐112 monomers binding to the Aβ42 seeds. This agrees with earlier reports that αSyn monomers bind Aβ42 fibrils [14], and our hypothesis that Aβ42 fibrils serve as a nucleation platform for αSyn. The largest increment in αSyn‐112 content in the pellets was observed at 4 days, which, again, is in accordance with the aggregation half‐time measured by ThT (Fig. 2B). Hence, the results from the kinetic experiments using ThT and the results obtained using SDS‐PAGE are fully consistent and thus corroborate the mechanism of αSyn‐Aβ co‐aggregation we put forward in this study.

### αSyn isoforms exhibit distinct aggregate morphologies in the presence of Aβ42

**Fig. 4:**
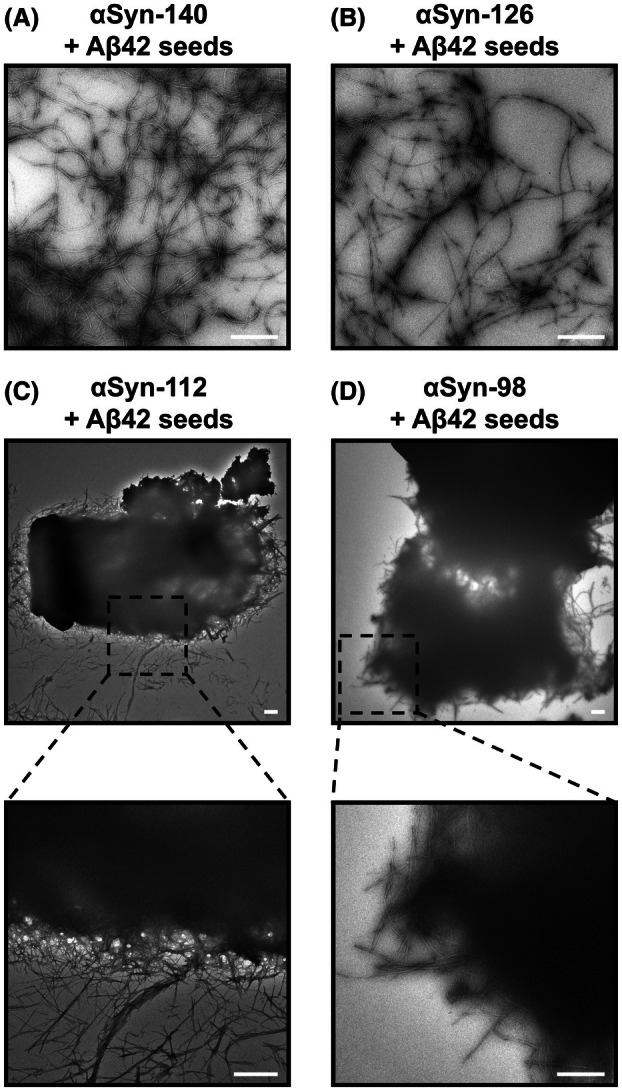
αSyn isoform aggregates formed in the presence of Aβ42 seeds are morphologically distinct. (A–D) Representative TEM images of αSyn isoform aggregates formed by incubating 100 μm αSyn‐140 (A), 50 μm αSyn‐126 (B), 50 μm αSyn‐112 (C) or 50 μm αSyn‐98 (D) monomers with 5 μm Aβ42 seeds. αSyn‐140 and αSyn‐126 aggregated into elongated amyloid fibrils (A, B), whereas αSyn‐112 and αSyn‐98 aggregated into clumps several micrometres in diameter, consisting of a fibrillar network (C, D). Fibrils can be visualised radiating out of these clumps, as shown in the representative images at higher magnification. All scale bars show 1 μm.

We next investigated whether the differences in aggregation kinetics between the αSyn isoforms (Figs 1 and 2) were also associated with the formation of morphologically distinct aggregates. We therefore imaged the samples from the aggregation reactions of αSyn isoform monomers with Aβ42 seeds (Fig. 2) using transmission electron microscopy (TEM) and showed that, in all instances, amyloid fibrils were formed (Fig. 4). Notably, the morphologies closely resemble those previously observed for these isoforms [34]. αSyn‐140 and αSyn‐126 aggregated into elongated amyloid fibrils, which form a thin layer on the grid (Fig. 4A,B), whereas αSyn‐112 and αSyn‐98 form dense aggregate clumps several micrometres in diameter. The latter consist of a network of amyloid fibrils, which can be visualised radiating at the edge (Fig. 4C,D). The sequestration of amyloid fibrils into larger clumps may be due to the isoelectric points of the αSyn‐112 and αSyn‐98 isoforms in the neutral range, allowing lateral association of the fibrils, as opposed to acidic isoelectric points for αSyn‐140 and αSyn‐126, rendering these isoforms negatively charged at neutral pH.

### The aggregation kinetics of αSyn‐140 depend on the ratio of Aβ42 seeds

**Fig. 5:**
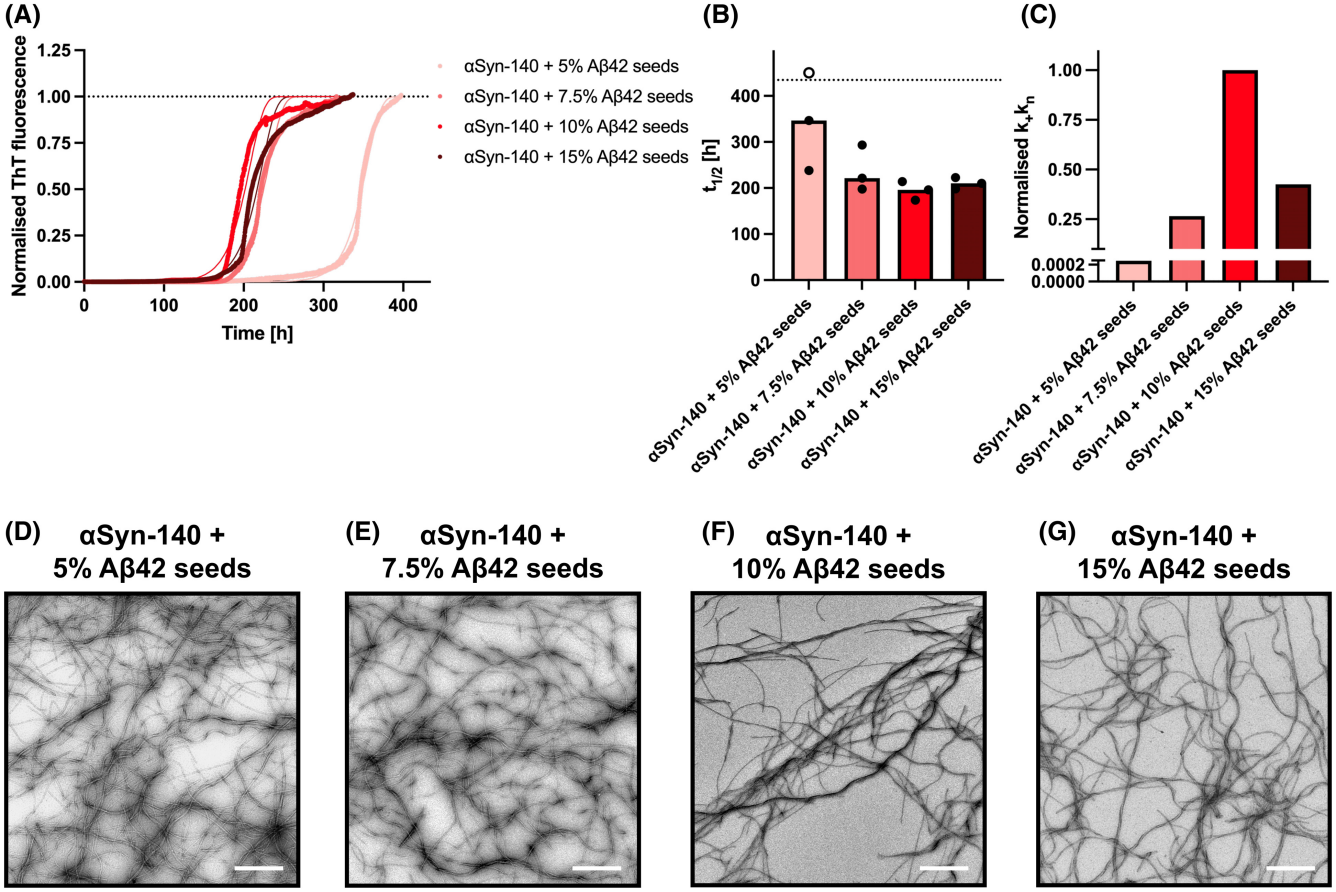
The ratio of Aβ42 seeds modulates the aggregation kinetics of αSyn‐140. (A) The aggregation of 75 μm αSyn‐140 with varying ratios of Aβ42 seeds was assessed by measuring ThT fluorescence intensity over time. Normalised aggregation traces were fitted with the saturating elongation and fragmentation model, shown as the solid lines, using the AmyloFit platform [39]. (B) Half‐times (*t*_1/2_) of the aggregation traces shown in (A). Data are shown as medians (bars) of three replicates (circles). If the aggregation reaction was completed, half‐times are shown as closed circles; otherwise, replicates are displayed as open circles at a value higher than the total run time. (C) Kinetic parameter k_+_k_n_ (primary nucleation and elongation) derived from the fits of the aggregation data in (A). (D–G) Representative TEM images of the aggregates formed during the aggregation reaction in (A). Elongated amyloid fibrils were observed in all cases. All scale bars show 1 μm.

Having established that Aβ42 seeds induce the aggregation of αSyn isoform monomers into amyloid fibrils, we sought to understand in more detail the extent to which the aggregation kinetics of αSyn depend on the amount of Aβ42 seeds serving as a nucleation site. Therefore, we varied the relative content of Aβ42 seeds from 5% to 15% (monomer equivalents) using a fixed concentration of the main αSyn‐140 isoform at a concentration of 75 μm (Fig. 5). We chose this concentration specifically as it was the lowest concentration of αSyn‐140 at which aggregation was observed (Fig. 2C). We found that increasing the Aβ42 seed content from 5% to 7.5% led to a strong acceleration of αSyn‐140 aggregation, reducing the half‐time by 36%, from 347 to 222 h (Fig. 5A,B). Further elevating the Aβ42 seed content from 7.5% to 10% only led to a minor acceleration, reducing the half‐time to 196 h. At this condition, the aggregation of αSyn‐140 with Aβ42 seeds appeared to be at its optimum as a further increase to 15% Aβ42 seeds conversely led to an increase in half‐time to 210 h. This effect may be due to enhanced adsorption of αSyn‐140 monomers to the surfaces of Aβ42 seeds, whereby fewer free monomers are available for primary nucleation. To model the accelerating effect of Aβ42 seeds on αSyn‐140 aggregation, we fitted the aggregation traces using the AmyloFit platform by varying the rate constant for primary nucleation and elongation, *k*_+_ *k*_n_, and maintaining all other kinetic parameters constant (Fig. 5A,C and Table S1) [39]. This revealed that an increase from 5% to 7.5% Aβ42 seeds increased the k_+_k_n_ value more than 1000‐fold, whereas a further increase to 10% Aβ42 seeds only elicited another 3.8‐fold increase of the k_+_k_n_ value. Adding 15% Aβ42 seeds, in turn, reduced the k_+_k_n_ value by 57% compared to mixtures with 10% Aβ42 seeds (Fig. 5C). Furthermore, we assessed that similar aggregates were formed while varying the Aβ42 seed content using TEM, showing elongated amyloid fibrils in all conditions (Fig. 5D–G).

### αSyn‐112 further accelerates the aggregation of αSyn‐140 in the presence of Aβ42 seeds

**Fig. 6:**
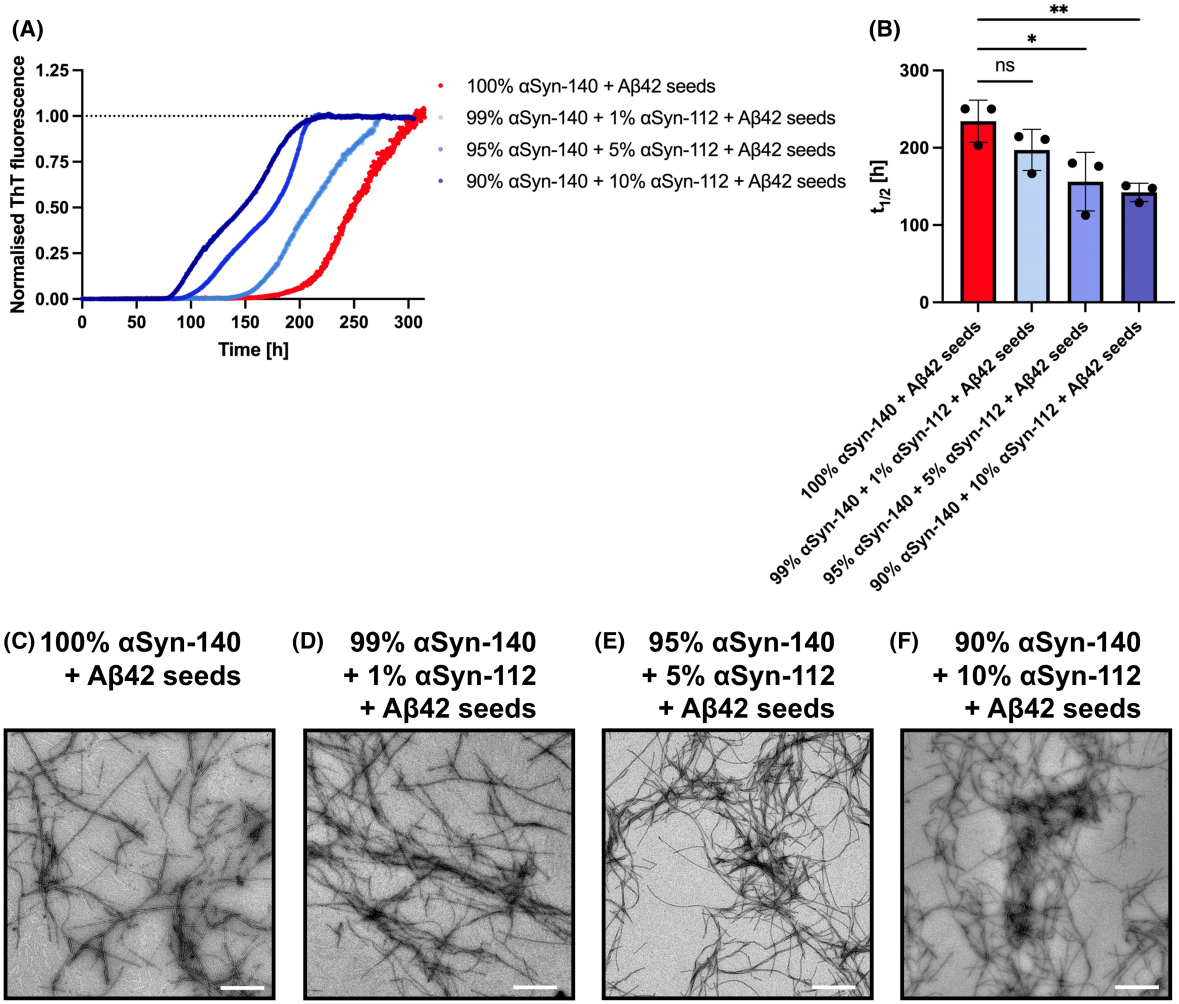
Co‐aggregation of αSyn‐140 with the αSyn‐112 isoform in the presence of Aβ42 seeds. (A) The aggregation of 100 μm αSyn with 5 μm Aβ42 seeds was assessed by measuring ThT fluorescence intensity over time. The αSyn content was composed of 100% αSyn‐140, 99% αSyn‐140 with 1% αSyn‐112, 95% αSyn‐140 with 5% αSyn‐112 or 90% αSyn‐140 with 10% αSyn‐112. (B) Half‐times (*t*_1/2_) of the aggregation traces shown in (A). Data are shown as means ± SD of three replicates. One‐way ANOVA with Dunnett's *post hoc* test. **P < 0.01, *P < 0.05, ns = non‐significant. (C–F) Representative TEM images of the aggregates formed during the aggregation reaction in (A). Elongated amyloid fibrils were observed in all cases. All scale bars show 1 μm.

So far, we explored how Aβ42 seeds induce the aggregation of αSyn isoforms, and that the relative aggregation propensities of αSyn isoforms and the morphologies of the formed aggregates vary depending on their sequence composition. Hence, we wondered whether a modulating effect of the alternative isoforms αSyn‐126, αSyn‐112 and αSyn‐98 on the main isoform αSyn‐140, which was reported previously [34], can also be observed when aggregation is induced by Aβ42 seeds. To test this prediction, we compared the aggregation of different mixtures of 100 μm αSyn monomers, where the αSyn content was either composed of 100% αSyn‐140 or 90% αSyn‐140 mixed with 10% of one of the three isoforms (namely αSyn‐126, αSyn‐112 or αSyn‐98). All of these mixtures were tested in the presence of 5 μm Aβ42 seeds (Fig. 6A,B and Fig. S6A,B). While 10% αSyn‐126 and αSyn‐98 had no significant effect on the overall aggregation, introducing 10% αSyn‐112 significantly reduced the aggregation half‐time by ~40%, from 234 h (100% αSyn‐140) to 142 h (90% αSyn‐140 + 10% αSyn‐112) (Fig. S6A,B).

An additional reason for primarily investigating αSyn‐112 is that this isoform possesses an enhanced aggregation propensity and was shown to significantly enhance the overall aggregation when added to an excess of αSyn‐140 [34]. Therefore, we further assessed the effects of even lower concentrations of αSyn‐112, mixing 95% αSyn‐140 with 5% αSyn‐112, and 99% αSyn‐140 with 1% αSyn‐112, in the presence of 5 μm Aβ42 seeds (Fig. 6A). We observed a trend towards accelerated aggregation at 1% αSyn‐112 (*t*_1/2_ = 197 h), which became statistically significant at 5% αSyn‐112 (*t*_1/2_ = 156 h) (Fig. 6B). Finally, we visualised the formed aggregates, showing that elongated amyloid fibrils are present in all conditions, whereas fibril clumps, as for αSyn‐112 or αSyn‐98, were no longer observed (Fig. 6C–F and Fig. S6C–F). This supports the notion that monomers of the alternative isoforms (αSyn‐126, αSyn‐112, αSyn‐98) were incorporated into fibrils with αSyn‐140 and that enhanced primary nucleation between αSyn‐140 and αSyn‐112, rather than the *in‐situ* formation of αSyn‐112 aggregates which cross‐seed the aggregation of αSyn‐140, is the main mechanism behind the aggregation process. We thus believe that this underlies the acceleration of the overall aggregation.

### αSyn isoform aggregates do not accelerate Aβ42 aggregation

Throughout this study, we have investigated the mechanism by which Aβ42, in its monomeric or pre‐aggregated form, affects the aggregation of four prominent αSyn isoforms. Given the frequent co‐pathologies observed between αSyn and Aβ, we also explored whether pre‐formed αSyn isoform aggregates could conversely affect the aggregation of Aβ42. To this end, we incubated 1.0 μm Aβ42 monomers with low concentrations (1–5%, i.e. 10–50 nm) or equimolar concentrations (100%, i.e. 1.0 μm) of pre‐formed aggregates of each of the four αSyn isoforms (Fig. S7A,C,E,G). However, the aggregation kinetics of Aβ42 remained mostly unaffected by the presence of αSyn isoform aggregates, and no acceleration of Aβ42 aggregation was observed across the conditions tested. Instead, we observed a slight increase in the aggregation half‐times with incremental addition of αSyn isoform seeds (Fig. S7B,D,F,H). The greatest effect on the aggregation half‐time was achieved by adding equimolar concentrations of αSyn‐140 (23% increase) and αSyn‐112 (26% increase) aggregates (Fig. S7B,F). These results support our notion that the more aggregation‐prone Aβ42 peptide undergoes self‐assembly first and that these Aβ42 aggregates drive the aggregation of αSyn isoforms, rather than αSyn isoforms driving Aβ42 aggregation.

## Discussion

In this study, we investigated the aggregation of the αSyn splice isoforms, αSyn‐140, αSyn‐126, αSyn‐112 and αSyn‐98, in the presence of Aβ42 in order to understand the molecular mechanisms and interactions between these proteins, which have increasingly been reported to form co‐pathologies in neurodegenerative disorders [17, 18]. We demonstrated that, in monomeric mixtures of Aβ42 with αSyn isoforms, Aβ42 can induce the aggregation of αSyn isoforms. This occurs because Aβ42 undergoes aggregation first, which is followed by the aggregation of αSyn isoforms (Fig. 1 and Fig. S3). Notably, concentrations as low as 0.1 μm of Aβ42 monomers were sufficient to promote the aggregation of αSyn isoforms (Fig. S4). This is particularly relevant as concentrations of Aβ peptides may reach up to micromolar levels when they are taken up by cells and enter the endolysosomal system [43, 44], which represents one of several possible pathways whereby Aβ can come into contact with αSyn.

We further tested whether a similar effect was observed when incubating αSyn isoforms with Aβ40 instead of Aβ42 monomers. The Aβ40 variant is the most abundant form of Aβ in the brain and is known to be significantly less aggregation‐prone compared to Aβ42 due to the lack of the two C‐terminal hydrophobic amino acids [45, 46]. We observed an aggregation profile similar to that with Aβ42, whereby Aβ40 monomers underwent aggregation first, as expected, significantly more slowly than Aβ42, followed by the aggregation of αSyn isoforms (Fig. S5). The only difference between Aβ40 and Aβ42 lies in the fact that no clear trend was observed with regard to the inhibition of the initial Aβ40 aggregation by αSyn isoforms. Consequently, the overall aggregation seemed to depend on the interplay of each individual αSyn isoform with Aβ40. This is in line with studies showing that the regions involved in the interaction with the main αSyn‐140 differ between Aβ42 and Aβ40 [47]. Together, these results corroborate our previous findings and demonstrate that different Aβ variants may act in promoting the aggregation of αSyn isoforms, which bears important implications for potential *in‐vivo* aggregation processes, where multiple Aβ variants are present simultaneously.

Hence, based on our above findings, a potential mechanism is that Aβ fibrils formed *in situ* facilitate the aggregation of αSyn isoforms. We confirmed this by showing that the aggregation of αSyn isoforms was further accelerated when pre‐formed Aβ42 seeds instead of Aβ42 monomers were added (Fig. 2). We further independently validated both of these observations using SDS‐PAGE, allowing us to evaluate which of the two proteins, αSyn or Aβ42, had aggregated at different timepoints of the reaction (Fig. 3). In contrast to another study [29], by monitoring extended timescales, we were thus able to show that Aβ42 seeds do, in fact, induce the aggregation of αSyn isoforms, including the less aggregation‐prone main αSyn‐140 isoform.

**Fig. 7:**
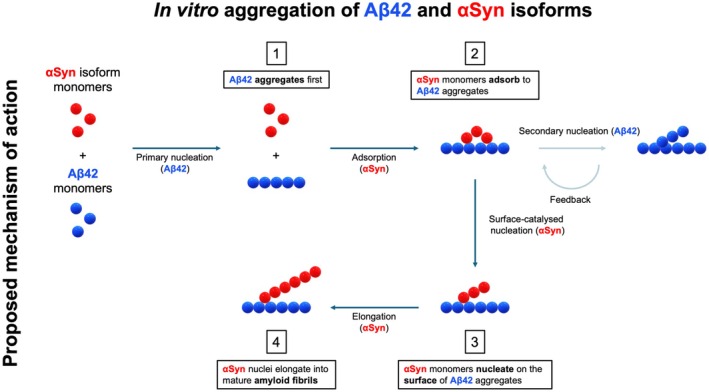
Mechanistic scheme of the microscopic processes involved in the co‐aggregation of Aβ42 and αSyn. Starting from a sample containing monomeric Aβ42 and αSyn, the initial event is the rapid formation of Aβ42 seeds, which then catalyse the much slower aggregation of αSyn. Notably, αSyn monomers inhibit the secondary nucleation of Aβ42, and the αSyn‐112 isoform further accelerates the aggregation of the αSyn‐140 isoform in the presence of Aβ42 seeds.

Thus, our results show that Aβ42 seeds act as nucleation surfaces for the aggregation of αSyn isoforms. This observation corroborates the idea that the aggregation of αSyn *in vitro* is an inherently slow process and is most commonly achieved by providing additional surfaces, such as lipid membranes, to which αSyn monomers can adsorb [12, 48, 49]. The resulting increase in local αSyn concentration facilitates its primary nucleation and thereby promotes αSyn aggregation. Thus, introducing stable surfaces reduces the aggregation lag time of αSyn isoforms. Similarly, Aβ42 seeds were shown to provide a surface for αSyn condensate formation under liquid‐liquid phase‐separating conditions [50]. This effect has been observed for other protein systems, including Aβ, where a change in lipid interface can significantly modulate the primary nucleation rate [49, 51]. Moreover, in recent years, there has been extensive research on amyloid oligomers, which are an intermediate species between native monomers and amyloid fibrils. Oligomers of Aβ and αSyn have been shown to exert neurotoxic effects by disrupting lipid membranes, forming pores and interfering with synaptic transmission, disrupting calcium homeostasis and inducing oxidative stress [52, 53, 54, 55, 56]. The formation of large fibrillar deposits has therefore been hypothesised to be a safety mechanism to clear neurotoxic oligomers [3, 7]. However, in the context of our study, we should note that we have not been able to accelerate the aggregation of αSyn with transient species such as oligomers [29]. Instead, in our experiments, the Aβ fibrils act as nucleation sites from which αSyn can aggregate. The fact that Aβ42 seeds offer nucleation surfaces directly links the aggregation processes of Aβ42 and αSyn. Hence, these results indicate a possible mechanism that may explain the frequent observation of Aβ and αSyn co‐deposition, by which the highly aggregation‐prone Aβ42 peptides form aggregates first, which in turn catalyse the aggregation of αSyn (Fig. 7).

Additionally, we were able to reveal key aspects of the aggregation behaviour of αSyn splice isoforms. Firstly, isoforms lacking the C‐terminal, highly negatively charged exon 5, that is αSyn‐112 and αSyn‐98, are more aggregation‐prone than isoforms containing exon 5, that is αSyn‐140 and αSyn‐126 (Figs 1 and 2). Secondly, the morphologies of the resulting amyloid aggregates were similar to those observed previously: while αSyn‐140 and αSyn‐126 formed elongated amyloid fibrils, αSyn‐112 and αSyn‐98 formed dense fibril clumps several micrometres in diameter (Fig. 4) [34]. Moreover, varying the content of Aβ42 seeds did not affect the morphology of the formed αSyn‐140 fibrils (Fig. 5), which in turn supports a mechanism of heterogeneous, surface‐catalysed nucleation [57]. Thirdly, adding small amounts of αSyn‐112 to αSyn‐140 in the presence of Aβ42 seeds significantly accelerated the aggregation kinetics of αSyn‐140 (Fig. 6). This finding further supports the idea that increased production of αSyn‐112 may be a pathogenic factor in the development of synucleinopathies [34].

The results of this study form part of a wider context exploring how co‐pathologies can occur in neurodegenerative diseases. Aβ and αSyn have been extensively reported to aggregate into amyloid fibrils, which are the major components of the hallmark amyloid plaques in AD and Lewy bodies in PD, respectively. However, it is becoming increasingly evident that Aβ and αSyn form co‐pathologies, with several studies indicating that about 50% of AD patients exhibit Lewy body pathology, and up to 80% of patients with Lewy body disease exhibit Aβ pathology upon *post‐mortem* analysis [18, 58, 59]. Importantly, these lesions were found to act in synergy, further exacerbating the disease progression [60]. Moreover, other amyloidogenic proteins have been shown to interact and co‐deposit in various constellations, thereby creating a spectrum of interrelated clinical conditions instead of distinct disorders. Besides Aβ and αSyn, amyloid proteins that have been reported to undergo co‐deposition include the microtubule‐associated protein tau, prion protein, huntingtin, the transactive response DNA‐binding protein 43 (TDP‐43) and fused in sarcoma (FUS) [18, 59, 61, 62, 63, 64]. These observations underlie the emerging concept of mixed proteinopathies, in which the formation of amyloid deposits is not limited to merely one protein but rather involves multiple different amyloidogenic proteins [18, 65]. In addition, it should be noted that, instead of a single molecular species, each of these proteins physiologically exists as a set of proteoforms that may be generated by alternative splicing, proteolytic truncations and chemical modifications. Thus, the combination of these processes drastically increases the number of possible interactions between the above‐mentioned proteins, further complicating the process [66, 67, 68].

Additionally, a series of biological factors have been reported to modulate the self‐assembly of misfolding proteins into amyloid structures. These include lipids, small‐molecule metabolites, metal ions, as well as peptides and other proteins [35, 51, 69, 70, 71, 72]. As part of this group, antioxidants have received increased attention recently as they are capable of both inhibiting amyloid aggregation as well as mitigating oxidative stress, which represents a potent mechanism contributing to cytotoxicity in amyloid‐burdened neurons [73, 74, 75]. To unravel the molecular basis of neurodegeneration and to develop disease‐modifying therapeutic strategies, it will be crucial to understand how to influence this intricate network of interactions between amyloid protein variants as well as with other biomolecules in a targeted manner.

In conclusion, we investigated the co‐aggregation mechanisms of four αSyn isoforms with Aβ42, providing key insights into the molecular processes that may underlie the frequently observed Aβ and αSyn co‐pathologies. Our findings also prompt further investigations into the role of alternative splicing in the pathogenic aggregation of αSyn. We elucidated the mechanisms that govern this co‐aggregation process and provided a kinetic framework explaining this synergistic effect. Integrating the findings that Aβ42 seeds act as a nucleation surface for αSyn aggregation together with the effects of alternative splicing on αSyn aggregation propensity may yield novel points of intervention against the pathogenesis of a range of devastating neurodegenerative disorders. By further building on these results, new therapeutic routes that specifically target these co‐aggregation mechanisms in the context of neurodegeneration may be developed.

## Author contributions

AR and ZT performed all the experiments. All authors analysed the data. ZT and MV supervised the project. All authors wrote the manuscript.

## Peer review

The peer review history for this article is available at https://www.webofscience.com/api/gateway/wos/peer‐review/10.1002/1873‐3468.70118.

## Supporting information


**Fig. S1.** Deconvoluted LC–MS spectra of purified proteins used in this study.
**Fig. S2.** Aggregation of αSyn isoforms in the absence of Aβ42.
**Fig. S3.** Normalised aggregation traces of αSyn isoforms in the presence of Aβ42 monomers.
**Fig. S4.** Aggregation of αSyn isoforms at lower Aβ42 concentrations.
**Fig. S5.** Aggregation of αSyn isoforms in the presence of Aβ40 monomers.
**Fig. S6.** Co‐aggregation of αSyn isoforms in the presence of Aβ42 seeds.
**Fig. S7.** Aggregation of Aβ42 in the presence of αSyn isoform aggregates.
**Table S1.** Kinetic parameters derived from varying Aβ42 seed contents.

## Data Availability

There are no restrictions on data and materials accessibility. The data that supports the findings of this study are available in all figures and the Supporting Information of this article.

## References

[1] Wimo A , Seeher K , Cataldi R , Cyhlarova E , Dielemann JL , Frisell O , Guerchet M , Jönsson L , Malaha AK and Nichols E (2023) The worldwide costs of dementia in 2019. Alzheimers Dement 19, 2865–2873. 36617519 10.1002/alz.12901 PMC10842637

[2] Livingston G , Huntley J , Liu KY , Costafreda SG , Selbæk G , Alladi S , Ames D , Banerjee S , Burns A and Brayne C (2024) Dementia prevention, intervention, and care: 2024 report of the lancet standing commission. Lancet 404, 572–628. 39096926 10.1016/S0140-6736(24)01296-0

[3] Knowles TP , Vendruscolo M and Dobson CM (2014) The amyloid state and its association with protein misfolding diseases. Nat Rev Mol Cell Biol 15, 384–396. 24854788 10.1038/nrm3810

[4] Jack CR Jr , Bennett DA , Blennow K , Carrillo MC , Dunn B , Haeberlein SB , Holtzman DM , Jagust W , Jessen F , Karlawish J *et al*. (2018) NIA‐AA research framework: toward a biological definition of Alzheimer's disease. Alzheimers Dement 14, 535–562. 29653606 10.1016/j.jalz.2018.02.018 PMC5958625

[5] Höglinger GU , Adler CH , Berg D , Klein C , Outeiro TF , Poewe W , Postuma R , Stoessl AJ and Lang AE (2024) A biological classification of Parkinson's disease: the SynNeurGe research diagnostic criteria. Lancet Neurol 23, 191–204. 38267191 10.1016/S1474-4422(23)00404-0

[6] Hampel H , Hardy J , Blennow K , Chen C , Perry G , Kim SH , Villemagne VL , Aisen P , Vendruscolo M , Iwatsubo T *et al*. (2021) The amyloid‐β pathway in Alzheimer's disease. Mol Psychiatry 26, 5481–5503. 34456336 10.1038/s41380-021-01249-0 PMC8758495

[7] Selkoe DJ and Hardy J (2016) The amyloid hypothesis of Alzheimer's disease at 25 years. EMBO Mol Med 8, 595–608. 27025652 10.15252/emmm.201606210 PMC4888851

[8] Spillantini MG , Schmidt ML , Lee VM‐Y , Trojanowski JQ , Jakes R and Goedert M (1997) α‐Synuclein in Lewy bodies. Nature 388, 839–840. 9278044 10.1038/42166

[9] Poewe W , Seppi K , Tanner CM , Halliday GM , Brundin P , Volkmann J , Schrag A‐E and Lang AE (2017) Parkinson disease. Nat Rev Dis Primers 3, 1–21. 10.1038/nrdp.2017.13 28332488

[10] Michaels TC , Qian D , Šarić A , Vendruscolo M , Linse S and Knowles TP (2023) Amyloid formation as a protein phase transition. Nat Rev Phys 5, 379–397.

[11] Habchi J , Chia S , Galvagnion C , Michaels TC , Bellaiche MM , Ruggeri FS , Sanguanini M , Idini I , Kumita JR and Sparr E (2018) Cholesterol catalyses Aβ42 aggregation through a heterogeneous nucleation pathway in the presence of lipid membranes. Nat Chem 10, 673–683. 29736006 10.1038/s41557-018-0031-x

[12] Galvagnion C , Buell AK , Meisl G , Michaels TC , Vendruscolo M , Knowles TP and Dobson CM (2015) Lipid vesicles trigger α‐synuclein aggregation by stimulating primary nucleation. Nat Chem Biol 11, 229–234. 25643172 10.1038/nchembio.1750 PMC5019199

[13] Linse S , Cabaleiro‐Lago C , Xue W‐F , Lynch I , Lindman S , Thulin E , Radford SE and Dawson KA (2007) Nucleation of protein fibrillation by nanoparticles. Proc Natl Acad Sci U S A 104, 8691–8696. 17485668 10.1073/pnas.0701250104 PMC1866183

[14] Chia S , Flagmeier P , Habchi J , Lattanzi V , Linse S , Dobson CM , Knowles TP and Vendruscolo M (2017) Monomeric and fibrillar α‐synuclein exert opposite effects on the catalytic cycle that promotes the proliferation of Aβ42 aggregates. Proc Natl Acad Sci U S A 114, 8005–8010. 28698377 10.1073/pnas.1700239114 PMC5544282

[15] Xia Z , Prescott EE , Urbanek A , Wareing HE , King MC , Olerinyova A , Dakin H , Leah T , Barnes KA and Matuszyk MM (2024) Co‐aggregation with apolipoprotein E modulates the function of amyloid‐β in Alzheimer's disease. Nat Commun 15, 4695. 38824138 10.1038/s41467-024-49028-z PMC11144216

[16] Wagner J , Degenhardt K , Veit M , Louros N , Konstantoulea K , Skodras A , Wild K , Liu P , Obermüller U , Bansal V *et al*. (2022) Medin co‐aggregates with vascular amyloid‐β in Alzheimer's disease. Nature 612, 123–131. 36385530 10.1038/s41586-022-05440-3 PMC9712113

[17] Galpern WR and Lang AE (2006) Interface between tauopathies and synucleinopathies: a tale of two proteins. Ann Neurol 59, 449–458. 16489609 10.1002/ana.20819

[18] Spires‐Jones TL , Attems J and Thal DR (2017) Interactions of pathological proteins in neurodegenerative diseases. Acta Neuropathol 134, 187–205. 28401333 10.1007/s00401-017-1709-7 PMC5508034

[19] Ueda K , Fukushima H , Masliah E , Xia Y , Iwai A , Yoshimoto M , Otero D , Kondo J , Ihara Y and Saitoh T (1993) Molecular cloning of cDNA encoding an unrecognized component of amyloid in Alzheimer disease. Proc Natl Acad Sci U S A 90, 11282–11286. 8248242 10.1073/pnas.90.23.11282 PMC47966

[20] Bachhuber T , Katzmarski N , McCarter JF , Loreth D , Tahirovic S , Kamp F , Abou‐Ajram C , Nuscher B , Serrano‐Pozo A and Müller A (2015) Inhibition of amyloid‐β plaque formation by α‐synuclein. Nat Med 21, 802–807. 26099047 10.1038/nm.3885

[21] Khan SS , LaCroix M , Boyle G , Sherman MA , Brown JL , Amar F , Aldaco J , Lee MK , Bloom GS and Lesné SE (2018) Bidirectional modulation of Alzheimer phenotype by alpha‐synuclein in mice and primary neurons. Acta Neuropathol 136, 589–605. 29995210 10.1007/s00401-018-1886-z PMC6329667

[22] Clinton LK , Blurton‐Jones M , Myczek K , Trojanowski JQ and LaFerla FM (2010) Synergistic interactions between aβ, tau, and α‐synuclein: acceleration of neuropathology and cognitive decline. J Neurosci 30, 7281–7289. 20505094 10.1523/JNEUROSCI.0490-10.2010 PMC3308018

[23] Lloyd GM , Dhillon J‐KS , Gorion K‐MM , Riffe C , Fromholt SE , Xia Y , Giasson BI and Borchelt DR (2021) Collusion of α‐synuclein and aβ aggravating co‐morbidities in a novel prion‐type mouse model. Mol Neurodegener 16, 1–17. 34503546 10.1186/s13024-021-00486-9 PMC8427941

[24] Bassil F , Brown HJ , Pattabhiraman S , Iwasyk JE , Maghames CM , Meymand ES , Cox TO , Riddle DM , Zhang B and Trojanowski JQ (2020) Amyloid‐beta (aβ) plaques promote seeding and spreading of alpha‐synuclein and tau in a mouse model of Lewy body disorders with aβ pathology. Neuron 105, 260–275.e266. 31759806 10.1016/j.neuron.2019.10.010 PMC6981053

[25] Jensen P , Sørensen E , Petersen T , Gliemann J and Rasmussen L (1995) Residues in the synuclein consensus motif of the α‐synuclein fragment, NAC, participate in transglutaminase‐catalysed cross‐linking to Alzheimer‐disease amyloid β A4 peptide. Biochem J 310, 91–94. 7646476 10.1042/bj3100091 PMC1135858

[26] Candreva J , Chau E , Rice ME and Kim JR (2019) Interactions between soluble species of β‐amyloid and α‐synuclein promote oligomerization while inhibiting fibrillization. Biochemistry 59, 425–435. 31854188 10.1021/acs.biochem.9b00655 PMC7269195

[27] Ono K , Takahashi R , Ikeda T and Yamada M (2012) Cross‐seeding effects of amyloid β‐protein and α‐synuclein. J Neurochem 122, 883–890. 22734715 10.1111/j.1471-4159.2012.07847.x

[28] Köppen J , Schulze A , Machner L , Wermann M , Eichentopf R , Guthardt M , Hähnel A , Klehm J , Kriegeskorte M‐C and Hartlage‐Rübsamen M (2020) Amyloid‐beta peptides trigger aggregation of alpha‐synuclein in vitro. Molecules 25, 580. 32013170 10.3390/molecules25030580 PMC7037551

[29] Vadukul DM , Papp M , Thrush RJ , Wang J , Jin Y , Arosio P and Aprile FA (2023) α‐Synuclein aggregation is triggered by oligomeric amyloid‐β 42 via heterogeneous primary nucleation. J Am Chem Soc 145, 18276–18285. 37556728 10.1021/jacs.3c03212 PMC10450681

[30] Campion D , Martin C , Heilig R , Charbonnier F , Moreau V , Flaman JM , Petit JL , Hannequin D , Brice A and Frebourg T (1995) The NACP/synuclein gene: chromosomal assignment and screening for alterations in Alzheimer disease. Genomics 26, 254–257. 7601450 10.1016/0888-7543(95)80208-4

[31] Beyer K , Domingo‐Sábat M , Lao JI , Carrato C , Ferrer I and Ariza A (2008) Identification and characterization of a new alpha‐synuclein isoform and its role in Lewy body diseases. Neurogenetics 9, 15–23. 17955272 10.1007/s10048-007-0106-0

[32] Gámez‐Valero A and Beyer K (2018) Alternative splicing of alpha‐and beta‐synuclein genes plays differential roles in synucleinopathies. Genes 9, 63. 29370097 10.3390/genes9020063 PMC5852559

[33] Röntgen A , Toprakcioglu Z , Dada ST , Morris OM , Knowles TP and Vendruscolo M (2025) Aggregation of α‐synuclein splice isoforms through a phase separation pathway. Sci Adv 11, eadq5396. 40238878 10.1126/sciadv.adq5396 PMC12002138

[34] Röntgen A , Toprakcioglu Z , Tomkins JE and Vendruscolo M (2024) Modulation of α‐synuclein in vitro aggregation kinetics by its alternative splice isoforms. Proc Natl Acad Sci U S A 121, e2313465121. 38324572 10.1073/pnas.2313465121 PMC10873642

[35] Dada ST , Toprakcioglu Z , Cali MP , Röntgen A , Hardenberg MC , Morris OM , Mrugalla LK , Knowles TP and Vendruscolo M (2024) Pharmacological inhibition of α‐synuclein aggregation within liquid condensates. Nat Commun 15, 3835. 38714700 10.1038/s41467-024-47585-x PMC11076612

[36] Abelein A , Chen G , Kitoka K , Aleksis R , Oleskovs F , Sarr M , Landreh M , Pahnke J , Nordling K and Kronqvist N (2020) High‐yield production of amyloid‐β peptide enabled by a customized spider silk domain. Sci Rep 10, 235. 31937841 10.1038/s41598-019-57143-x PMC6959368

[37] Shobo A , Röntgen A , Hancock MA and Multhaup G (2022) Biophysical characterization as a tool to predict amyloidogenic and toxic properties of amyloid‐β42 peptides. FEBS Lett 596, 1401–1411. 35466397 10.1002/1873-3468.14358

[38] Morris OM , Toprakcioglu Z , Röntgen A , Cali M , Knowles TP and Vendruscolo M (2024) Aggregation of the amyloid‐β peptide (Aβ40) within condensates generated through liquid–liquid phase separation. Sci Rep 14, 22633. 39349560 10.1038/s41598-024-72265-7 PMC11442885

[39] Meisl G , Kirkegaard JB , Arosio P , Michaels TC , Vendruscolo M , Dobson CM , Linse S and Knowles TP (2016) Molecular mechanisms of protein aggregation from global fitting of kinetic models. Nat Protoc 11, 252–272. 26741409 10.1038/nprot.2016.010

[40] Cohen SI , Linse S , Luheshi LM , Hellstrand E , White DA , Rajah L , Otzen DE , Vendruscolo M , Dobson CM and Knowles TP (2013) Proliferation of amyloid‐β42 aggregates occurs through a secondary nucleation mechanism. Proc Natl Acad Sci U S A 110, 9758–9763. 23703910 10.1073/pnas.1218402110 PMC3683769

[41] Habchi J , Chia S , Limbocker R , Mannini B , Ahn M , Perni M , Hansson O , Arosio P , Kumita JR , Challa PK *et al*. (2017) Systematic development of small molecules to inhibit specific microscopic steps of Aβ42 aggregation in Alzheimer's disease. Proc Natl Acad Sci U S A 114, E200–E208. 28011763 10.1073/pnas.1615613114 PMC5240708

[42] Hashimoto M , Bogdanovic N , Volkmann I , Aoki M , Winblad B and Tjernberg LO (2010) Analysis of microdissected human neurons by a sensitive ELISA reveals a correlation between elevated intracellular concentrations of Aβ42 and Alzheimer's disease neuropathology. Acta Neuropathol 119, 543–554. 20198479 10.1007/s00401-010-0661-6

[43] Hu X , Crick SL , Bu G , Frieden C , Pappu RV and Lee J‐M (2009) Amyloid seeds formed by cellular uptake, concentration, and aggregation of the amyloid‐beta peptide. Proc Natl Acad Sci U S A 106, 20324–20329. 19910533 10.1073/pnas.0911281106 PMC2787156

[44] Esbjörner EK , Chan F , Rees E , Erdelyi M , Luheshi LM , Bertoncini CW , Kaminski CF , Dobson CM and Schierle GSK (2014) Direct observations of amyloid β self‐assembly in live cells provide insights into differences in the kinetics of aβ (1–40) and aβ (1–42) aggregation. Chem Biol 21, 732–742. 24856820 10.1016/j.chembiol.2014.03.014 PMC4067742

[45] Näslund J , Schierhorn A , Hellman U , Lannfelt L , Roses AD , Tjernberg LO , Silberring J , Gandy SE , Winblad B and Greengard P (1994) Relative abundance of Alzheimer a beta amyloid peptide variants in Alzheimer disease and normal aging. Proc Natl Acad Sci U S A 91, 8378–8382. 8078890 10.1073/pnas.91.18.8378 PMC44609

[46] Meisl G , Yang X , Hellstrand E , Frohm B , Kirkegaard JB , Cohen SI , Dobson CM , Linse S and Knowles TP (2014) Differences in nucleation behavior underlie the contrasting aggregation kinetics of the Aβ40 and Aβ42 peptides. Proc Natl Acad Sci U S A 111, 9384–9389. 24938782 10.1073/pnas.1401564111 PMC4084462

[47] Kim JR (2023) Oligomerization by co‐assembly of β‐amyloid and α‐synuclein. Front Mol Biosci 10, 1153839. 37021111 10.3389/fmolb.2023.1153839 PMC10067735

[48] Fusco G , De Simone A , Gopinath T , Vostrikov V , Vendruscolo M , Dobson CM and Veglia G (2014) Direct observation of the three regions in α‐synuclein that determine its membrane‐bound behaviour. Nat Commun 5, 3827. 24871041 10.1038/ncomms4827 PMC4046108

[49] Toprakcioglu Z , Kamada A , Michaels TC , Xie M , Krausser J , Wei J , Saric A , Vendruscolo M and Knowles TP (2022) Adsorption free energy predicts amyloid protein nucleation rates. Proc Natl Acad Sci U S A 119, e2109718119. 35901206 10.1073/pnas.2109718119 PMC9351353

[50] Röntgen A , Toprakcioglu Z , Morris OM and Vendruscolo M (2025) Amyloid‐β modulates the phase separation and aggregation of α‐synuclein. Proceedings of the National Academy of Sciences 122(29), e2501987122, 10.1073/pnas.2501987122. PMC12305028 40658845

[51] Toprakcioglu Z , Jayaram AK and Knowles TP (2025) Ganglioside lipids inhibit the aggregation of the Alzheimer's amyloid‐β peptide. RSC Chem Biol 6, 809–822. 40109301 10.1039/d4cb00189c PMC11915136

[52] Fusco G , Chen SW , Williamson PT , Cascella R , Perni M , Jarvis JA , Cecchi C , Vendruscolo M , Chiti F , Cremades N *et al*. (2017) Structural basis of membrane disruption and cellular toxicity by α‐synuclein oligomers. Science 358, 1440–1443. 29242346 10.1126/science.aan6160

[53] Cascella R , Chen SW , Bigi A , Camino JD , Xu CK , Dobson CM , Chiti F , Cremades N and Cecchi C (2021) The release of toxic oligomers from α‐synuclein fibrils induces dysfunction in neuronal cells. Nat Commun 12, 1814. 33753734 10.1038/s41467-021-21937-3 PMC7985515

[54] Perni M , Mannini B , Xu CK , Kumita JR , Dobson CM , Chiti F and Vendruscolo M (2021) Exogenous misfolded protein oligomers can cross the intestinal barrier and cause a disease phenotype in *C. elegans* . Sci Rep 11, 14391. 34257326 10.1038/s41598-021-93527-8 PMC8277765

[55] Kayed R , Head E , Thompson JL , McIntire TM , Milton SC , Cotman CW and Glabe CG (2003) Common structure of soluble amyloid oligomers implies common mechanism of pathogenesis. Science 300, 486–489. 12702875 10.1126/science.1079469

[56] Haass C and Selkoe DJ (2007) Soluble protein oligomers in neurodegeneration: lessons from the Alzheimer's amyloid β‐peptide. Nat Rev Mol Cell Biol 8, 101–112. 17245412 10.1038/nrm2101

[57] Hadi Alijanvand S , Peduzzo A and Buell AK (2021) Secondary nucleation and the conservation of structural characteristics of amyloid fibril strains. Front Mol Biosci 8, 669994. 33937341 10.3389/fmolb.2021.669994 PMC8085410

[58] Hamilton RL (2000) Lewy bodies in Alzheimer's disease: a neuropathological review of 145 cases using α‐synuclein immunohistochemistry. Brain Pathol 10, 378–384. 10885656 10.1111/j.1750-3639.2000.tb00269.x PMC8098522

[59] Robinson JL , Lee EB , Xie SX , Rennert L , Suh E , Bredenberg C , Caswell C , Van Deerlin VM , Yan N and Yousef A (2018) Neurodegenerative disease concomitant proteinopathies are prevalent, age‐related and APOE4‐associated. Brain 141, 2181–2193. 29878075 10.1093/brain/awy146 PMC6022546

[60] Marsh SE and Blurton‐Jones M (2012) Examining the mechanisms that link β‐amyloid and α‐synuclein pathologies. Alzheimer's Res Ther 4, 11. 22546279 10.1186/alzrt109 PMC4054672

[61] Gomes LA , Hipp SA , Rijal Upadhaya A , Balakrishnan K , Ospitalieri S , Koper MJ , Largo‐Barrientos P , Uytterhoeven V , Reichwald J and Rabe S (2019) Aβ‐induced acceleration of Alzheimer‐related τ‐pathology spreading and its association with prion protein. Acta Neuropathol 138, 913–941. 31414210 10.1007/s00401-019-02053-5

[62] Sengupta U and Kayed R (2022) Amyloid β, tau, and α‐synuclein aggregates in the pathogenesis, prognosis, and therapeutics for neurodegenerative diseases. Prog Neurobiol 214, 102270. 35447272 10.1016/j.pneurobio.2022.102270

[63] St‐Amour I , Turgeon A , Goupil C , Planel E and Hébert SS (2018) Co‐occurrence of mixed proteinopathies in late‐stage Huntington's disease. Acta Neuropathol 135, 249–265. 29134321 10.1007/s00401-017-1786-7

[64] Scarioni M , Gami‐Patel P , Peeters CF , de Koning F , Seelaar H , Mol MO , van Swieten JC , Bank NB , Rozemuller AJ and Hoozemans JJ (2023) Psychiatric symptoms of frontotemporal dementia and subcortical (co‐) pathology burden: new insights. Brain 146, 307–320. 35136978 10.1093/brain/awac043 PMC9825544

[65] Forrest SL and Kovacs GG (2023) Current concepts of mixed pathologies in neurodegenerative diseases. Can J Neurol Sci 50, 329–345. 35356856 10.1017/cjn.2022.34

[66] Evans JR , Gustavsson EK , Doykov I , Murphy D , Virdi GS , Lachica J , Röntgen A , Murtada MH , Pang CW , Macpherson H *et al*. (2024) The diversity of SNCA transcripts in neurons, and its impact on antisense oligonucleotide therapeutics. *bioRxiv*. doi: 10.1101/2024.05.30.596437

[67] Wildburger NC , Esparza TJ , LeDuc RD , Fellers RT , Thomas PM , Cairns NJ , Kelleher NL , Bateman RJ and Brody DL (2017) Diversity of amyloid‐beta proteoforms in the Alzheimer's disease brain. Sci Rep 7, 9520. 28842697 10.1038/s41598-017-10422-x PMC5572664

[68] Beyer K and Ariza A (2013) Alpha‐synuclein posttranslational modification and alternative splicing as a trigger for neurodegeneration. Mol Neurobiol 47, 509–524. 22923347 10.1007/s12035-012-8330-5

[69] Biza KV , Nastou KC , Tsiolaki PL , Mastrokalou CV , Hamodrakas SJ and Iconomidou VA (2017) The amyloid interactome: exploring protein aggregation. PLoS One 12, e0173163. 28249044 10.1371/journal.pone.0173163 PMC5383009

[70] Joshi P , Chia S , Yang X , Perni M , Gabriel JM , Gilmer M , Limbocker R , Habchi J and Vendruscolo M (2023) Combinations of vitamin a and vitamin E metabolites confer resilience against amyloid‐β aggregation. ACS Chem Nerosci 14, 657–666. 10.1021/acschemneuro.2c00523 PMC9936541 36728544

[71] Shobo A , James N , Dai D , Röntgen A , Black C , Kwizera J‐R , Hancock MA , Bui KH and Multhaup G (2022) The amyloid‐β1–42‐oligomer interacting peptide D‐AIP possesses favorable biostability, pharmacokinetics, and brain region distribution. J Biol Chem 298, 101483. 34896396 10.1016/j.jbc.2021.101483 PMC8752909

[72] Giasson BI , Lee VM and Trojanowski JQ (2003) Interactions of amyloidogenic proteins. Neuromolecular Med 4, 49–58. 14528052 10.1385/NMM:4:1-2:49

[73] Ono K , Hamaguchi T , Naiki H and Yamada M (2006) Anti‐amyloidogenic effects of antioxidants: implications for the prevention and therapeutics of Alzheimer's disease. Biochim Biophys Acta 1762, 575–586. 16644188 10.1016/j.bbadis.2006.03.002

[74] Ali J , Choe K , Park JS , Park HY , Kang H , Park TJ and Kim MO (2024) The interplay of protein aggregation, genetics, and oxidative stress in Alzheimer's disease: role for natural antioxidants and immunotherapeutics. Antioxidants 13, 862. 39061930 10.3390/antiox13070862 PMC11274292

[75] Wang Y , Yan C , Qi Y , Zhao R , Wan Y , Lyu Z , Li Y , Wang Z , Li C and Lin Y (2025) Harnessing multi‐target nanosweepers inhibiting β‐amyloid aggregation, scavenging reactive oxygen species and overcoming the blood brain barrier for rescuing Alzheimer's disease. Chem Eng J 508, 161138.

