# Supplementary material for: Aβ42 promotes the aggregation of α‐synuclein splice isoforms via heterogeneous nucleation
Source: FEBS Lett. 2025 Jul 26;599(19):2750–67. doi: 10.1002/1873-3468.70118 (PMC12519046; doi:10.1002/1873-3468.70118)
Supplement: Supplementary file 1 — Fig. S1. Deconvoluted LC–MS spectra of purified proteins used in this study. Fig. S2. Aggregation of αSyn isoforms in the absence of Aβ42. Fig. S3. Normalised aggregation traces of αSyn isoforms in the presence of Aβ42 monomers. Fig. S4. Aggregation of αSyn isoforms at lower Aβ42 concentrations. Fig. S5. Aggregation of αSyn isoforms in the presence of Aβ40 monomers. Fig. S6. Co‐aggregation of αSyn isoforms in the presence of Aβ42 seeds. Fig. S7. Aggregation of Aβ42 in the presence of αSyn isoform aggregates. Table S1. Kinetic parameters derived from varying Aβ42 seed contents. [file FEB2-599-2750-s001.docx]

**SUPPORTING INFORMATION**

**Aβ42 promotes the aggregation of α-synuclein splice isoforms via heterogeneous nucleation**

Alexander Röntgen, Zenon Toprakcioglu^*^, and Michele Vendruscolo^*^

*Centre for Misfolding Diseases, Yusuf Hamied Department of Chemistry,*

*University of Cambridge, Cambridge CB2 1EW, United Kingdom*

**Correspondence to: zt231@cam.ac.uk, mv245@cam.ac.uk*

**
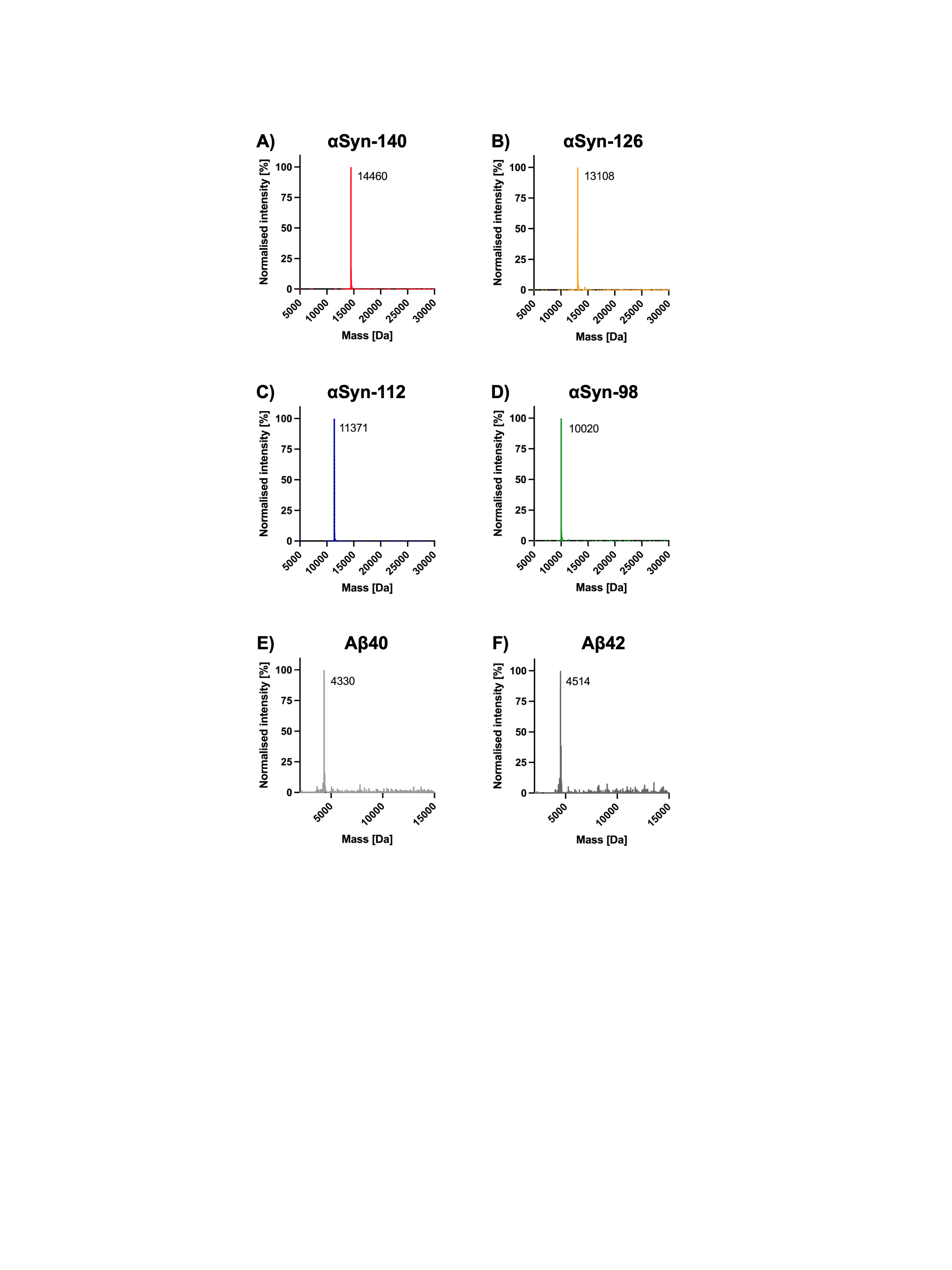
**

**Figure S1. Deconvoluted LC-MS spectra of purified proteins used in this study.**

**
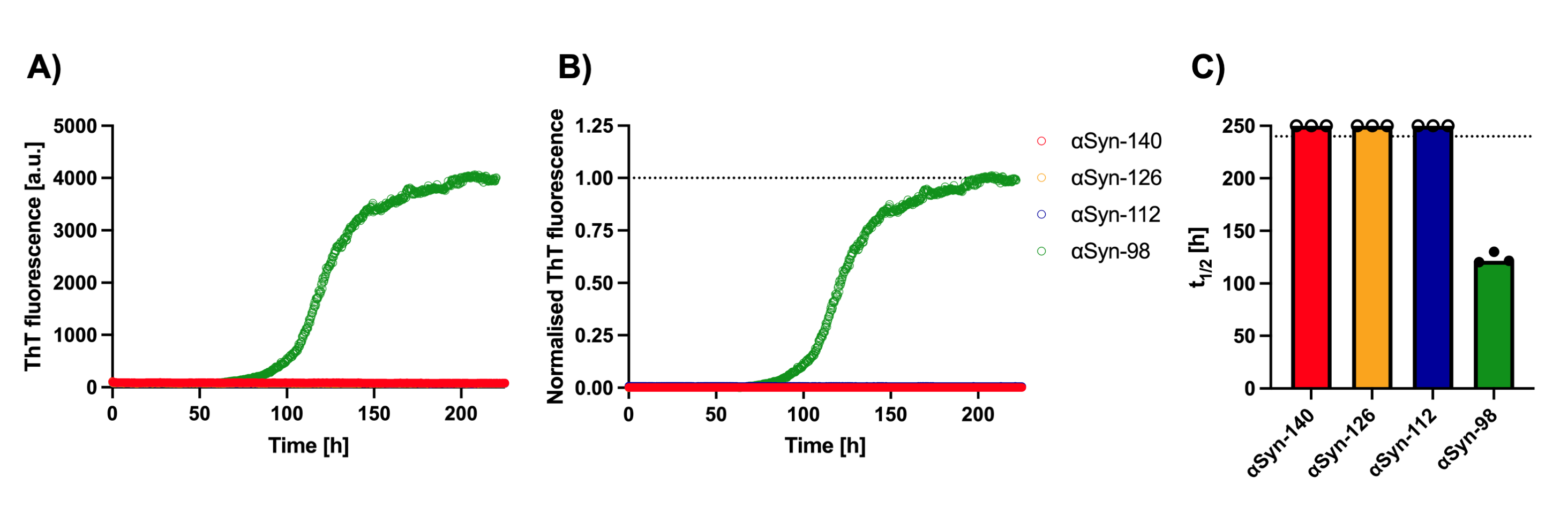
**

**Figure S2. Aggregation of αSyn isoforms in the absence of Aβ42. (A)** The aggregation of αSyn isoforms at 50 µM concentration was assessed over time by monitoring ThT fluorescence intensity. **(B)** Normalised aggregation traces shown in (A). **(C)** Half-times (t_1/2_) of the aggregation traces shown in (A,B). Data are shown as medians (bars) of three replicates (circles). If the aggregation was completed, half-times are shown as closed circles, otherwise replicates are displayed as open circles at a value higher than the total run time.

**
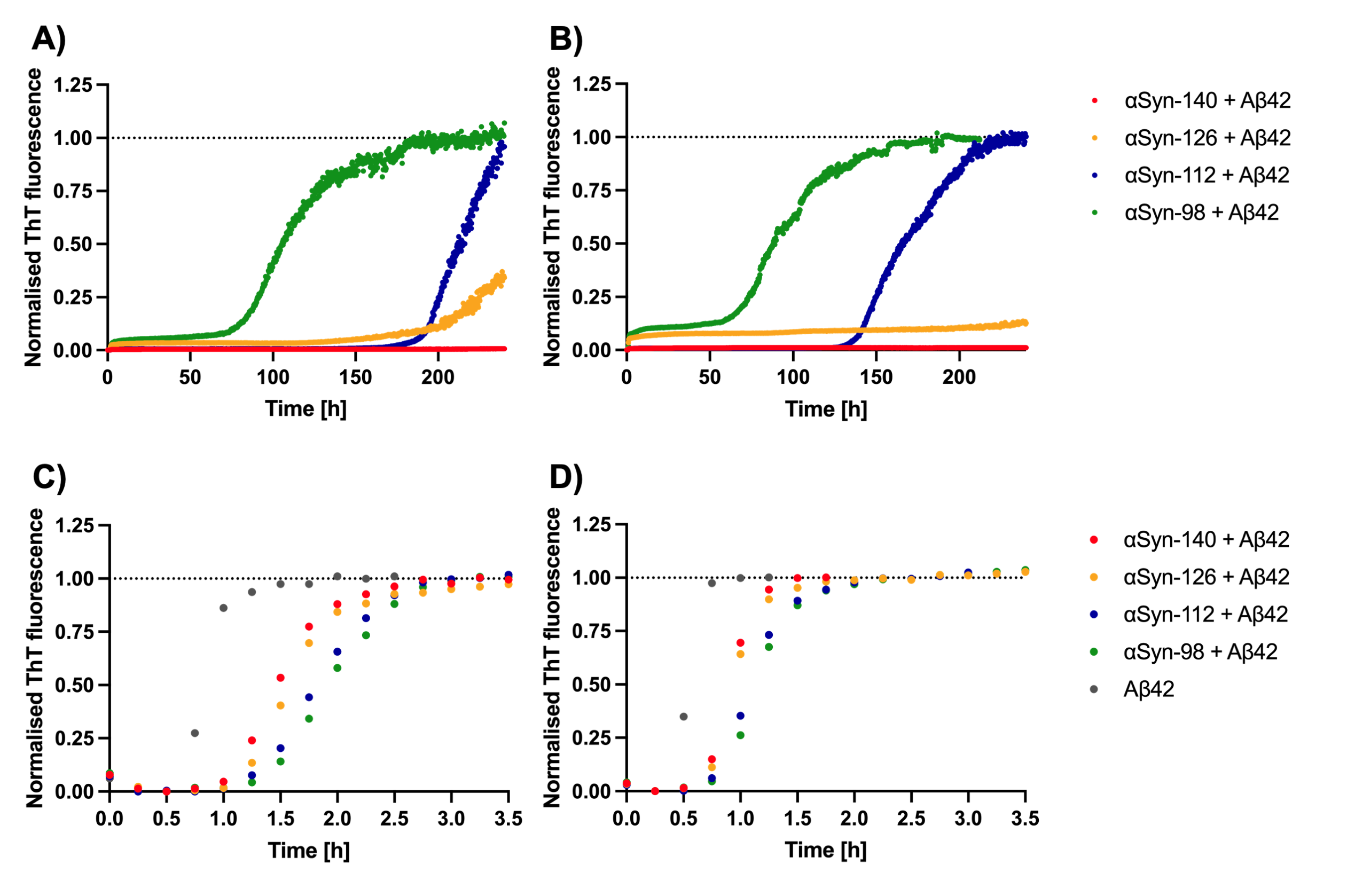
**

**Figure S3. Normalised aggregation traces of αSyn isoforms in the presence of Aβ42 monomers.** **(A,B)** The aggregation of αSyn isoforms with 2.5 μM Aβ42 (A) and 5.0 μM Aβ42 (B) at a total protein concentration of 50 μM was assessed over time by monitoring ThT fluorescence intensity. **(C,D)** Data of the first 3.5 h of the aggregation reactions showing the inhibition of Aβ42 aggregation by αSyn monomers before reaching the initial plateau phase.


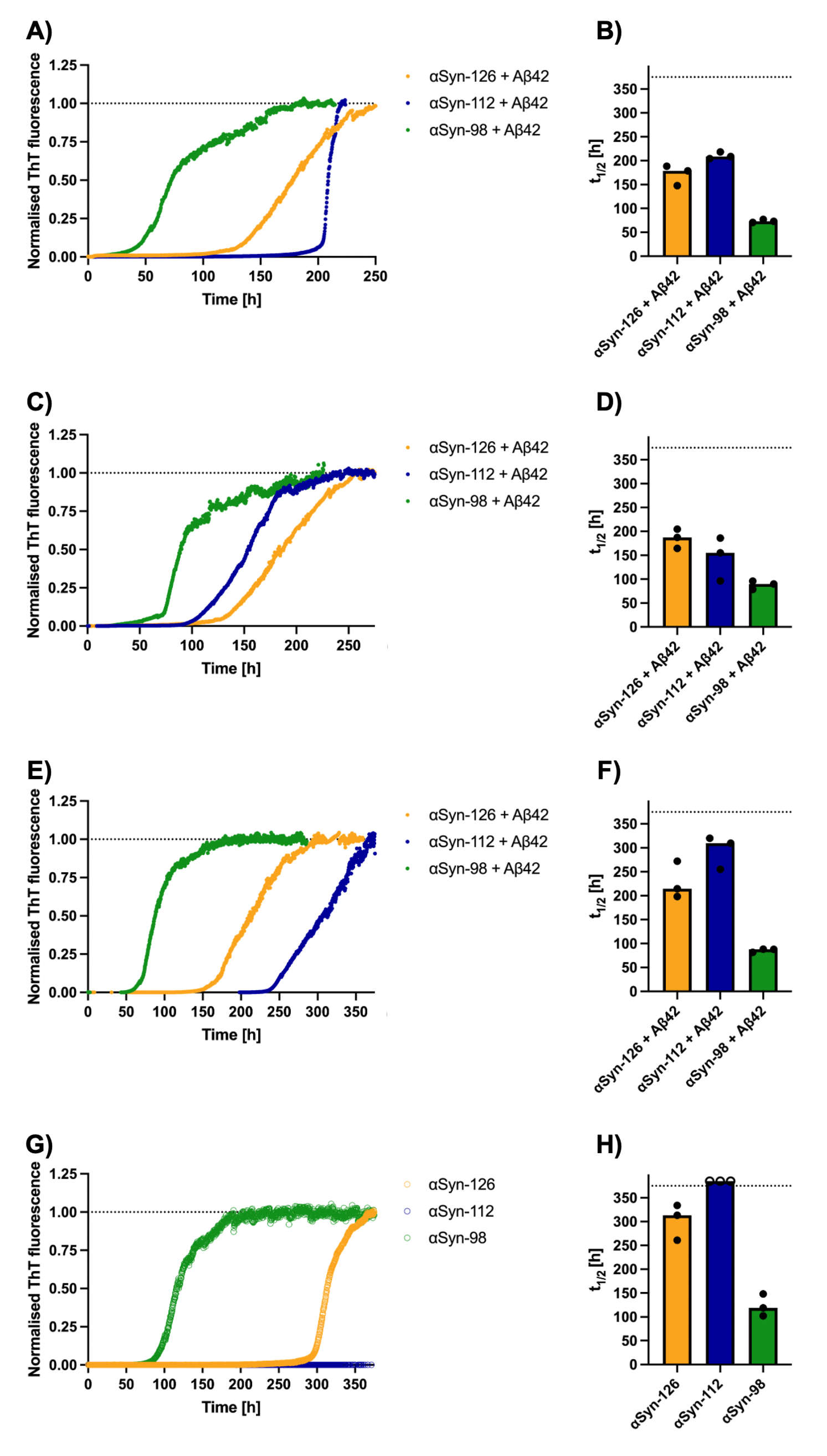


**Figure S4. Aggregation of αSyn isoforms at lower Aβ42 concentrations. (A,C,E,G)** The aggregation of 50 µM αSyn isoforms with 1.0 µM (A), 0.5 µM (C), 0.1 µM (E), or in the absence (G) of Aβ42 monomers was assessed over time by monitoring ThT fluorescence intensity. **(B,D,F,H)** Half-times (t_1/2_) of the aggregation traces shown in (A,C,E,G), respectively. Data are shown as medians (bars) of three replicates (circles). If the aggregation was completed, half-times are shown as closed circles, otherwise replicates are displayed as open circles at a value higher than the total run time.


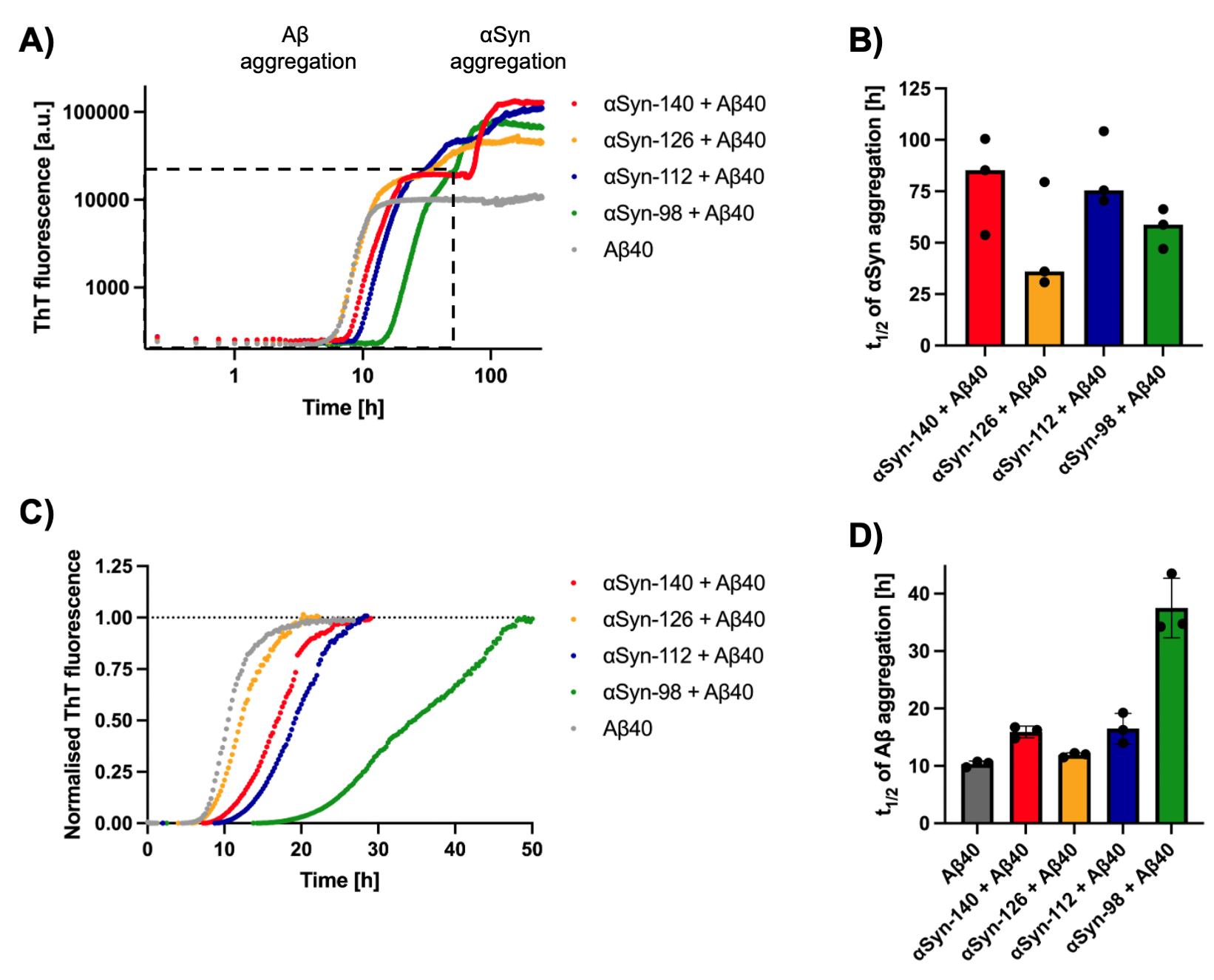


**Figure S5. Aggregation of αSyn isoforms in the presence of Aβ40 monomers. (A)** The aggregation of 50 µM αSyn isoforms with 5.0 µM of Aβ40 monomers was assessed over time by monitoring ThT fluorescence intensity. It should be noted that, under the conditions used, Aβ40 aggregates exhibit a significantly higher absolute ThT fluorescence than Aβ42 aggregates, as can be seen by comparing the control traces in Figure 1B and Figure S5A. **(B)**Half-times (t_1/2_) of the aggregation traces shown in (A). Data are shown as medians (bars) of three replicates (circles). **(C)** Magnification of the first 50 h of the aggregation reactions, indicated by the dashed rectangle in (A), showing the inhibition of Aβ40 aggregation by αSyn monomers. **(D)** Half-times (t_1/2_) of Aβ40 aggregation shown in (C). Data are shown as mean ± SD of three replicates.


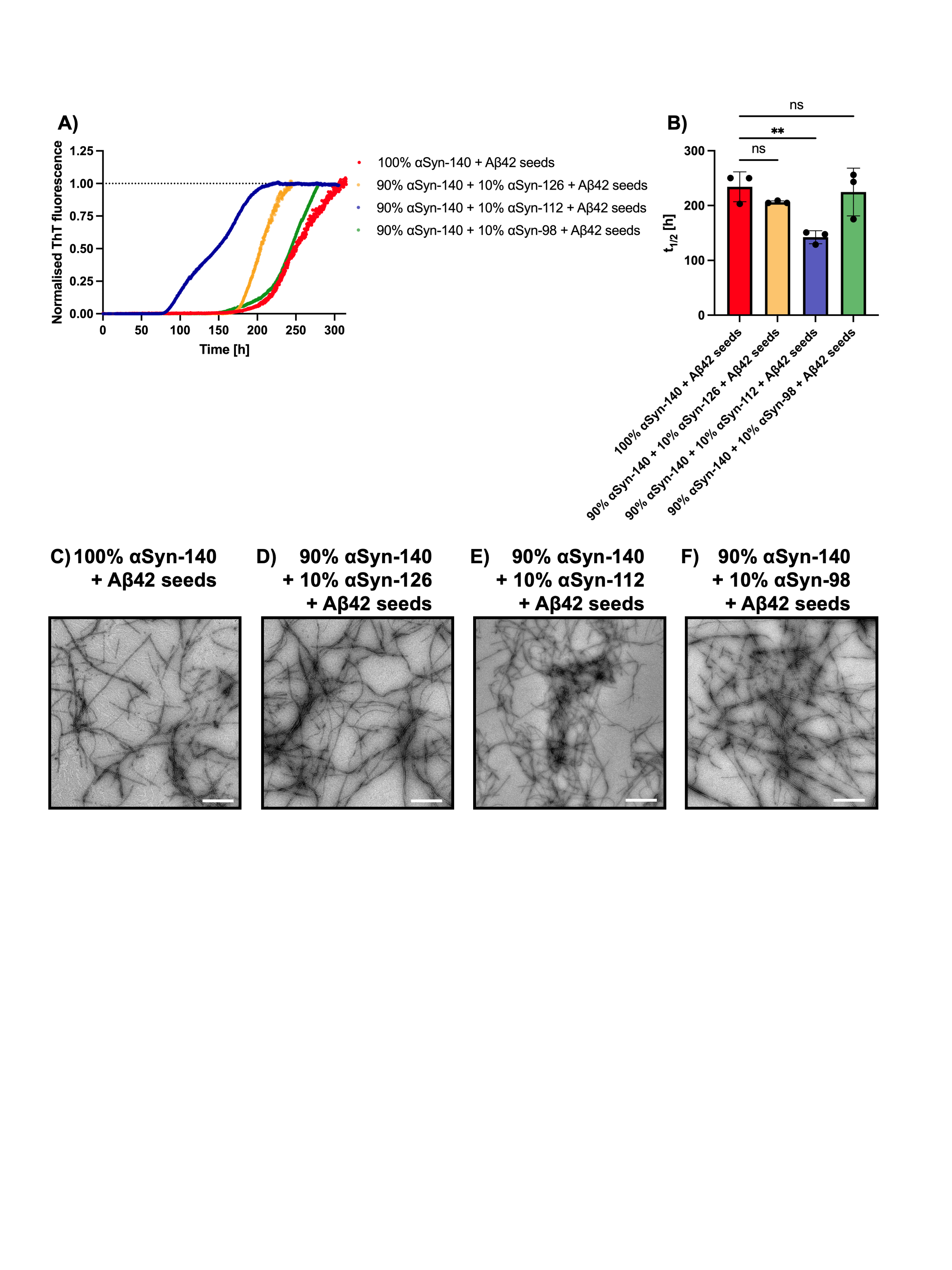


**Figure S6. Co-aggregation of αSyn isoforms in the presence of Aβ42 seeds. (A)** The aggregation of 100 μM αSyn with 5 μM Aβ42 seeds was assessed by measuring ThT fluorescence intensity over time. The αSyn content was composed of 100% αSyn-140 or 90% αSyn-140 with 10% αSyn-126, αSyn-112 or αSyn-98. **(B)** Half-times (t_1/2_) of the aggregation traces shown in (A). Data are shown as means ± SD of three replicates. One-way ANOVA with Dunnett’s post-hoc test. **p<0.01, ns = non-significant. **(C-F)** Representative TEM images of the aggregates formed during the aggregation reaction in (A). Elongated amyloid fibrils were observed in all cases. All scale bars show 1 μm. Data for 100% αSyn-140 and 90% αSyn-140 with 10% αSyn-112 are identical to Figure 5 since they were assessed together for comparison.

**
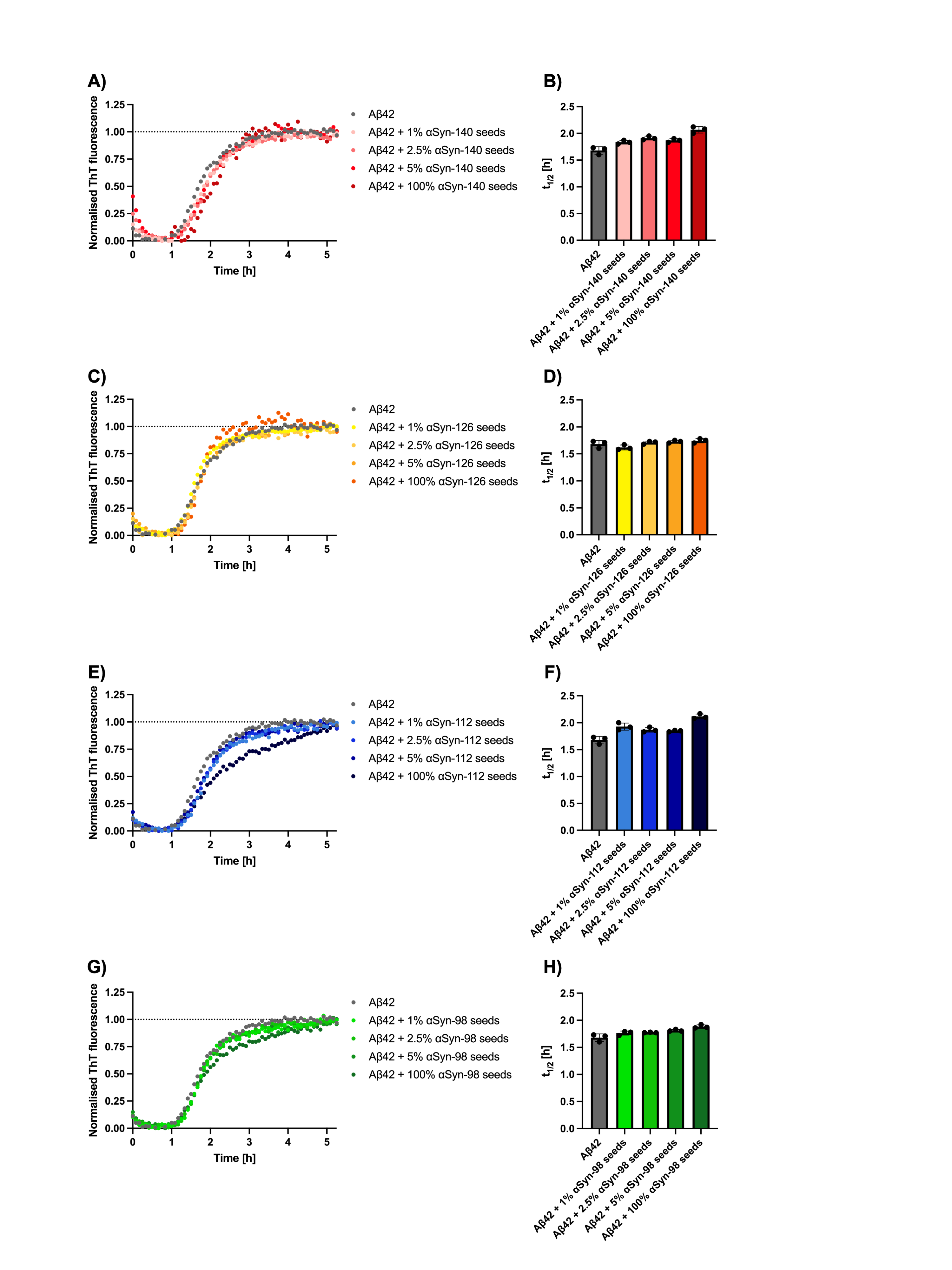
**

**Figure S7. Aggregation of Aβ42 in the presence of αSyn isoform aggregates.** **(A,C,E,G)** The aggregation of 1 µM Aβ40 with varying concentrations of αSyn isoform aggregates (1%–100%, *i.e.* 10 nM–1 µM) was assessed over time by monitoring ThT fluorescence intensity. **(B,D,F,H)** Half-times (t_1/2_) of the aggregation traces shown in (A,C,E,G). Data are shown as means ± SD of three replicates.

**Table S1. Kinetic parameters derived from varying Aβ42 seed contents.**

|  | **k_+_k_n_ [M-2 h^-2^]** | **k_+_k_–_ [M^-1^ h^-2^]** | **K_E_ [M]** | **n_c_** |
| --- | --- | --- | --- | --- |
| **5% Aβ42 seeds** | 1.06x10^–3^ | 21.4 | 4.81x10^4^ | 2 |
| **7.5% Aβ42 seeds** | 1.14 | 21.4 | 4.81x10^4^ | 2 |
| **10% Aβ42 seeds** | 4.30 | 21.4 | 4.81x10^4^ | 2 |
| **15% Aβ42 seeds** | 1.83 | 21.4 | 4.81x10^4^ | 2 |
